# Epitope-focused immunogen design based on the ebolavirus glycoprotein HR2-MPER region

**DOI:** 10.1371/journal.ppat.1010518

**Published:** 2022-05-18

**Authors:** Clara T. Schoeder, Pavlo Gilchuk, Amandeep K. Sangha, Kaitlyn V. Ledwitch, Delphine C. Malherbe, Xuan Zhang, Elad Binshtein, Lauren E. Williamson, Cristina E. Martina, Jinhui Dong, Erica Armstrong, Rachel Sutton, Rachel Nargi, Jessica Rodriguez, Natalia Kuzmina, Brooke Fiala, Neil P. King, Alexander Bukreyev, James E. Crowe, Jens Meiler

**Affiliations:** 1 Department of Chemistry, Vanderbilt University, Nashville, Tennessee, United States of America; 2 Center for Structural Biology, Vanderbilt University, Nashville, Tennessee, United States of America; 3 Institute for Drug Discovery, University Leipzig Medical School, Leipzig, Germany; 4 Vanderbilt Vaccine Center, Vanderbilt University Medical Center, Tennessee, United States of America; 5 Department of Pathology, University of Texas Medical Branch, Galveston, Texas, United States of America; 6 Galveston National Laboratory, Galveston, Texas, United States of America; 7 Department of Pathology, Microbiology, and Immunology, Vanderbilt University Medical Center, Nashville, Tennessee, United States of America; 8 Department of Biochemistry, University of Washington, Seattle, Washington, United States of America; 9 Institute for Protein Design, University of Washington, Seattle, Washington, United States of America; 10 Department of Microbiology & Immunology, University of Texas Medical Branch, Galveston, Texas, Unites States, United States of America; 11 Departments of Pediatrics, Vanderbilt University Medical Center, Nashville, Tennessee, United States of America; Friedrich-Loeffler-Institut, GERMANY

## Abstract

The three human pathogenic ebolaviruses: Zaire (EBOV), Bundibugyo (BDBV), and Sudan (SUDV) virus, cause severe disease with high fatality rates. Epitopes of ebolavirus glycoprotein (GP) recognized by antibodies with binding breadth for all three ebolaviruses are of major interest for rational vaccine design. In particular, the heptad repeat 2 –membrane-proximal external region (HR2-MPER) epitope is relatively conserved between EBOV, BDBV, and SUDV GP and targeted by human broadly-neutralizing antibodies. To study whether this epitope can serve as an immunogen for the elicitation of broadly-reactive antibody responses, protein design in Rosetta was employed to transplant the HR2-MPER epitope identified from a co-crystal structure with the known broadly-reactive monoclonal antibody (mAb) BDBV223 onto smaller scaffold proteins. From computational analysis, selected immunogen designs were produced as recombinant proteins and functionally validated, leading to the identification of a sterile alpha motif (SAM) domain displaying the BDBV-HR2-MPER epitope near its C terminus as a promising candidate. The immunogen was fused to one component of a self-assembling, two-component nanoparticle and tested for immunogenicity in rabbits. Robust titers of cross-reactive serum antibodies to BDBV and EBOV GPs and moderate titers to SUDV GP were induced following immunization. To confirm the structural composition of the immunogens, solution NMR studies were conducted and revealed structural flexibility in the C-terminal residues of the epitope. Overall, our study represents the first report on an epitope-focused immunogen design based on the structurally challenging BDBV-HR2-MPER epitope.

## Author summary

Recent breakthroughs in structure-based and computational-guided vaccine design have made methods available for transplanting epitopes from native antigens onto smaller scaffold proteins. We employed these methods to design epitope-focused immunogens based on the ebolavirus HR2-MPER epitope, which is targeted by potently neutralizing and broadly-reactive antibodies. Our efforts resulted in an immunogen that bound with high affinity to monoclonal antibodies and elicited high titers of epitope-specific polyclonal antibodies following rabbit immunization but failed to induce neutralizing antibodies. Structural characterization of the grafted epitope confirmed the overall immunogen model but also highlighted the inherent flexibility and complexity of the HR2-MPER epitope. This study demonstrates the current capabilities and remaining challenges of computational epitope-focused vaccine design. It also represents the first study of epitope-focused immunogen design based on an ebolavirus.

## Introduction

Ebolavirus disease (EVD) is caused in humans by three viruses of the genus *Ebolavirus*: Ebola virus (EBOV), being the most common; Bundibugyo virus (BDBV), which was first described in 2007 [1]; and the less phylogenetically related Sudan virus (SUDV). Although a vaccine was approved for EBOV in 2019, its development was based on EBOV GP sequences and it is not indicated for protection against BDBV and SUDV [2, 3].

Ebolaviruses and marburgviruses belong to the *Filovirus* family, named after the filamentous appearance of the virus in electron micrographs, and contain similarly structured genomes and protein compositions. A surface GP of the class I fusion protein family mediates host cell attachment and fusion and is the major target of the protective humoral immune response [4]. The GP is a trimer in which each protomer has two subunits: a GP1 head that is heavily glycosylated and a GP2 subunit that comprises the fusion machinery [5].

The human antibody response against ebolavirus GPs has been characterized in depth, and major antigenic targets of the humoral response have been identified and characterized functionally and structurally [6–10]. However, only few of the identified epitopes evoke antibody responses that recognize all three human pathogenic ebolaviruses, although many serological studies have shown occurrence of cross-reactivity at least for EBOV, BDBV and SUDV.[11, 12] These epitopes include at least two non-overlapping internal fusion loop epitopes, the GP1-core, the GP1-2 interface, and the HR2-MPER epitope. In contrast, many other epitopes are virus-specific, such as those in the glycan cap and mucin-like domain [6, 12, 13]. Epitopes targeted by cross-reactive antibodies are of major interest for rational vaccine design.

One class of neutralizing antibodies identified from survivors of the 2007 BDBV outbreak in Uganda targets the conserved HR2-MPER epitope [7]. This epitope consists of a linear peptide sequence in the GP2 subunit of the viral GP, framed by a N-terminal glycosylation site and in close proximity to the C-terminal transmembrane region of the protein. The HR2-MPER targeting antibody, BDBV223, shows extraordinary binding breadth across all three human pathogenic viruses and neutralizes BDBV and EBOV. In immunization studies using the BDBV-HR2-MPER peptide conjugated to keyhole limpet hemocyanin (KLH), the antigen elicited GP-reactive and neutralizing antibodies in animal serum with varying levels of neutralizing activity against EBOV, BDBV, and SUDV [14].

Rational, structure-based vaccine design has made enormous progress in the past decade. In particular, epitope-focused immunogen design transplants a specific epitope of interest onto a scaffold protein for display in a non-native environment. This allows for the targeting of a specific antibody or an antibody population with the goal of eliciting humoral responses focused on epitopes targeted by protective antibodies. Computational protein design methods are employed during this technique to ensure the epitope maintains the correct backbone and side chain conformations [15, 16]. A number of methods and protocols in the protein design software Rosetta, such as focusing on the transfer of sidechain residues involved in the interaction between antibody and antigen [17], the transfer of the whole backbone of either a linear or discontinuous epitope [18, 19] and the transfer of flexible regions such as loops [16, 20], have resulted in a number of antigens successfully targeted by single antibody populations. To reliably design a new immunogen, a high-resolution co-crystal structure of the epitope-antibody interface is necessary.

An apo-structure of BDBV223 and a peptide-antibody complex containing the BDBV-HR2-MPER epitope has been determined at a resolution of 2.03Å and 3.68 Å, respectively, by King et al. [21] providing the information required for structure-based immunogen design. Antibody recognition of the HR2-MPER epitope nevertheless remains enigmatic since the observed binding mode of the HR2-MPER peptide and BDBV223 is inaccessible to the antibody at most times due to its spatial localization close to the viral membrane [21]. In addition, HR2-MPER-reactive antibodies are low in abundance in immune subjects [22]. Similar epitopes have been described in other class I fusion proteins, *e*.*g*., the HIV envelope glycoprotein or SARS-CoV-2, where antibody recognition patterns raise the same questions [23, 24]. Nevertheless, designed proteins presenting solely the epitope could enhance the immune response to the HR2-MPER epitope.

The display of engineered antigens on self-assembling nanoparticles can enhance the induction of epitope-specific antibodies in immunization studies. Antigens including respiratory syncytial virus (RSV) prefusion-stabilized fusion (F) protein, human immunodeficiency virus (HIV) envelope glycoprotein, and the SARS-CoV-2 receptor binding domain have been displayed on computationally designed self-assembling protein nanoparticles, leading to significant increases in neutralizing potency and protective breadth [25–28]. Thus, a combination of epitope-focused immunogens and multivalent display on nanoparticle scaffolds ensures induction of high antibody titers [29].

Here, we explored epitope-focused immunogen design using epitope grafting protocols in Rosetta for the HR2-MPER epitope, as defined by the co-crystal structure of BDBV223 with the BDBV-HR2-MPER peptide (PDB: 6N7J) [21]. We tested designs experimentally for their ability to elicit antibody responses to the BDBV-HR2-MPER epitope and structurally validated the immunogen model through biomolecular nuclear magnetic resonance (NMR) spectroscopy.

## Results

### Rosetta epitope grafting protocols were used to design small protein immunogens carrying the HR2-MPER epitope

**Fig 1:**
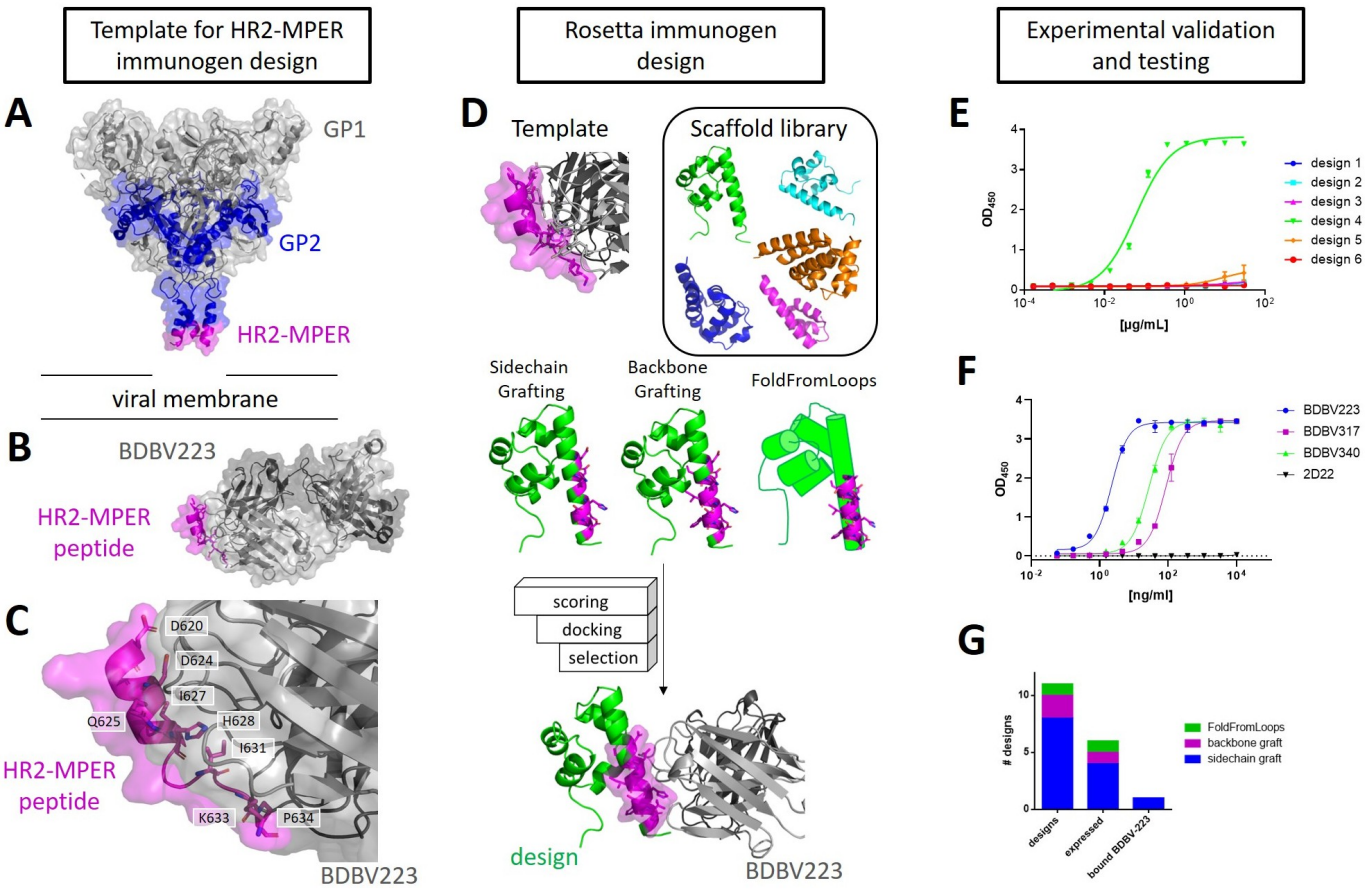
Computational design and initial experimental validation of the BDBV-MPER based immunogens. (A) BDBV GP, as observed in PDB: 6DZM [32], contains the soluble parts of GP1 and GP2, but not the HR2-MPER region. (B) Monoclonal antibody BDBV223 bound to the BDBV-MPER peptide (PDB: 6N7J) [21]. (C) A close-up view of the interactions of BDBV-GP with the BDBV-MPER peptide via critical interaction residues. (D) Rosetta grafting protocols are employed over a scaffold library containing small, highly resolved proteins, to transfer the epitope through Rosetta’s grafting protocols sidechain and backbone grafting, and FoldFromLoops [16, 18, 33]. (E) Out of eleven selected designs, six designs expressed from *E*. *coli* were tested for binding to BDBV223. Design 4, a sidechain graft of the epitope residues on the crystal structure PDB 1B0X, strongly bound to BDBV223 in ELISA (n = 2, duplicates, exemplary experiment plotted with standard deviation (SD); reference EC_50_ value for BDBV223 binding to the BDBV-MPER peptide has been reported as 85 ng/ml [14]; for further reference curves compare S1 Fig). (F) Binding of all three known BDBV-MPER targeting antibodies BDBV223, BDBV317 and BDBV340, to the BDBV-MPER carrying immunogen, while the control antibody, 2D22, a dengue E protein targeting antibody [34], does not bind to the immunogen (n = 2, duplicates, exemplary experiment plotted with SD). (G) Designs from sidechain grafting were the most prominent selected group tested. The selected design based on FoldFromLoops and one of the selected designs from backbone grafting were expressed but did not bind. The binding immunogen was one out of eleven designs.

The Rosetta software suite contains different methods for epitope-focused immunogen design, including sidechain and backbone grafting, and a protocol called FoldFromLoops, which is now succeeded by FunFolDes [16, 18, 20, 29, 30]. In the work presented here, these grafting protocols were used to transplant the BDBV-HR2-MPER epitope, as observed in the crystal structure PDB: 6N7J, onto smaller scaffold proteins [21]. In this co-crystal structure, BDBV223 was crystallized in complex with a synthetic peptide containing the linear BDBV-HR2-MPER epitope to a resolution of 3.68 Å. The BDBV-HR2-MPER peptide forms an α helix that interacts especially via residues D621, D624, H628, I631, and K633 with BDBV223 (Fig 1A, 1B and 1C). It is noteworthy that the C-terminal residues diverge from α-helical angles and the helical fold unravels. A glycan borders the N-terminal end of the HR2-MPER epitope in the full-length ebolavirus GP, which is not included in this structure. The Protein Database (PDB) was screened for small proteins with a resolution < 2.5 Å and reported to be expressible from *E*. *coli* for transplantation of the BDBV-HR2-MPER epitope from the co-crystal structure (PDB: 6N7J). Extensive filtering and screening steps were included, such as measures of protein stability, interaction with the BDBV223 crystal structure, and forward folding to ensure high quality of the designs (Fig 1D). In total, eleven designs were chosen for experimental validation. Designs were derived from different approaches, consisted of diverse sequences, and contained a total of three different scaffold proteins. In the case of the SAM domain (PDB: 1B0X) [31], additional refinement steps were undertaken to remove the dimer interface of the original crystal structure and help stabilize the monomeric protein. In total, one designed protein was chosen from a FoldFromLoops protocol run, two from backbone grafting, and eight from sidechain grafting (compare Fig 1F and S1 and S2 Tables for detailed protocol descriptions and designed sequences).

The designed proteins were expressed in *E*. *coli*, purified from the soluble fraction of lysed cells, and tested for binding by BDBV223. Out of the eleven selected designed proteins, six designs were purified. However, only one design bound to BDBV223 in ELISA (Fig 1E). The binding was specific for BDBV-HR2-MPER antibodies BDBV223, BDBV317 and BDBV340, but did not bind to 2D22, a dengue virus-specific antibody [34] (Fig 1F). This designed immunogen, designated BDBV-MPER, was derived from a *Mus muculus* SAM domain, which was observed in its native crystal structure as dimer [31] and was modified in order to remove interactions from the dimer interface for stabilization of the monomeric protein.

### The BDBV-MPER-based immunogen binds strongly to three HR2-MPER epitope-specific antibodies BDBV223, BDBV317, and BDBV340

**Fig 2:**
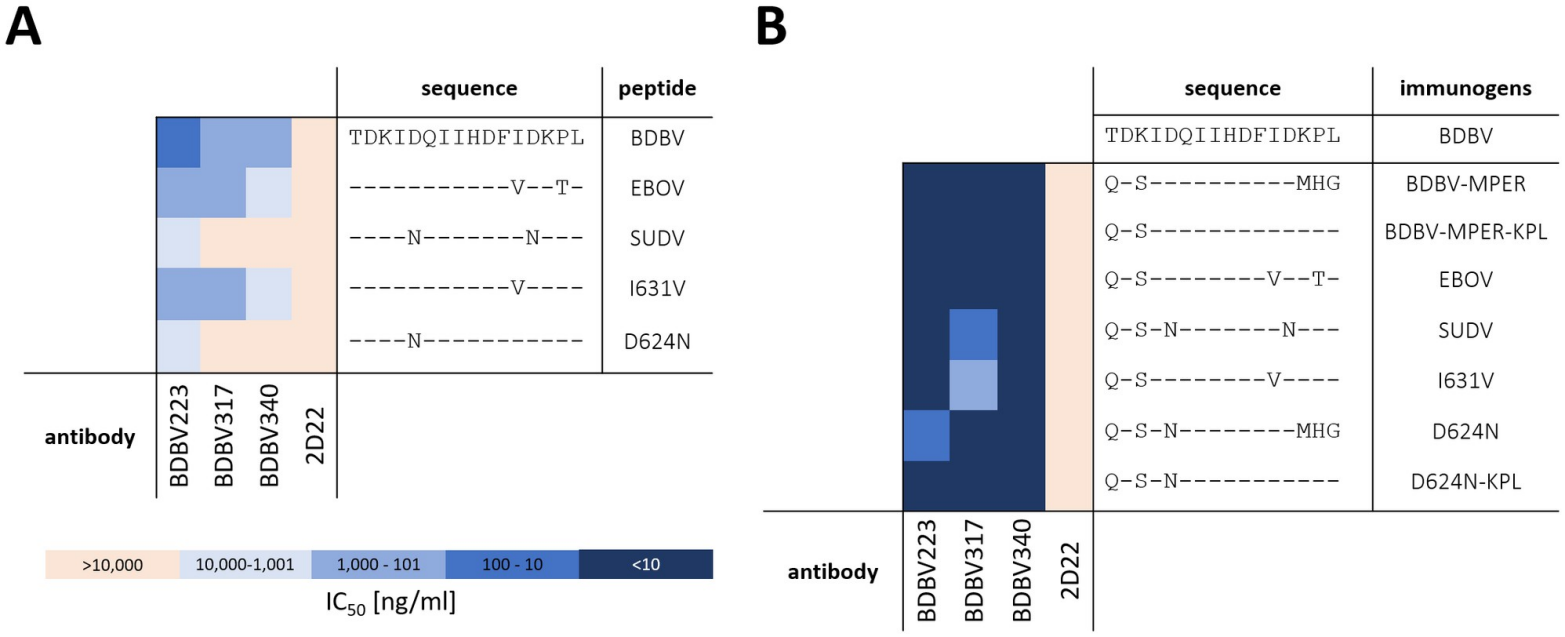
Antibody binding of BDBV223, BDBV317 and BDBD340 to (A) HR2-MPER peptides (data taken from Flyak et al. [14]) or to (B) designed immunogens carrying HR2-MPER sequences of BDBV, SUDV and EBOV GP or mutants to characterize sequence activity relationships. 2D22, a dengue antibody [34], was used as control for non-specific binding, in ELISA. Peptides were coated at a concentration of 4 μg/mL, whereas immunogens were used at 1 μg/mL, n = 2, duplicates (S1 Fig).

The designed immunogen incorporated residues I623 to D632 of the BDBV-HR2-MPER epitope. As observed in the crystal structure, residues K633 and P634 contact BDBV223 [21]. Therefore, we ensured that the designed immunogen recapitulated the known sequence-activity relationships that were reported by Flyak et al. [14] through introduction of the same mutations in our immunogens (Fig 2A). Using site-directed mutagenesis, the three C-terminal residues MHG were replaced with the last three residues of the BDBV-HR2-MPER epitope, specifically with the residues KPL (designated BDBV-MPER-KPL). Additionally, immunogens carrying the sequence of the SUDV- and EBOV-HR2-MPER were studied. Through ELISA binding, we observed strong dose-response binding for the three HR2-MPER antibodies (BDBV223, BDBV317 and BDBV340) to the BDBV-MPER and BDBV-MPER-KPL immunogens. In comparison to the native BDBV-HR2-MPER epitope sequence, both BDBV-MPER and BDBV-MPER-KPL immunogens contain a different starting amino acid in the third position (glutamate instead of a threonine and a serine instead of a lysine), which in the crystal structure PBD 6N7J failed to form any interactions with BDBV223 [21], and should not impact the binding properties of the immunogen. The last three amino acids in the BDBV-MPER immunogen, however, showed interactions with the antibody in the crystal structure. As the impact of these changes was unclear, we assessed the binding properties of both the BDBV-MPER and BDBV-MPER-KPL immunogens. Binding was comparable, with half maximal effective concentration (EC_50_) values of <1 ng/mL, suggesting the last three amino acids minimally contribute to the binding interaction (Fig 2).

For peptides derived from the BDBV-HR2-MPER sequence, a binding pattern was recently reported by Flyak et al. [14] for the three antibodies BDBV223, BDBV317 and BDBV340. All three antibodies revealed strongest binding to the homologous BDBV-HR2-MPER peptide, bound less strongly to the EBOV-HR2-MPER peptide, and showed reduced or no binding to the SUDV-HR2-MPER peptide [14]. In order to investigate this loss of binding, we tested a BDBV-HR2-MPER-D624N immunogen, which contains one of the two amino acid changes of the SUDV-HR2-MPER peptide to test the hypothesis that BDBV223 binds differentially compared to BDBV317 and BDBV340 [14].

Immunogens carrying the sequences for SUDV-, EBOV-HR2-MPER, and respective mutations were expressed, purified, and assessed by ELISA. BDBV223, BDBV317 and BDBV340 bound strongly with EC_50_ values of < 10 ng/mL to BDBV-MPER, BDBV-MPER-KPL, or EBOV-MPER immunogens, showing no preference for the BDBV-HR2-MPER epitope sequences compared to the native EBOV-HR2-MPER sequence. BDBV317 exhibited weaker binding to the SUDV-MPER immunogen, but still displayed an EC_50_ value < 100 ng/mL. When introducing mutations to the immunogen similar to the mutations studied for HR2-MPER peptides [14], only the BDBV-MPER-I631V mutant diminished binding to BDBV317. For antibody BDBV223 and BDBV340, the binding potency for all immunogens stayed the same (S1 Fig).

### Multivalent display on self-assembling nanoparticle platform for enhanced immune recognition

**Fig 3:**
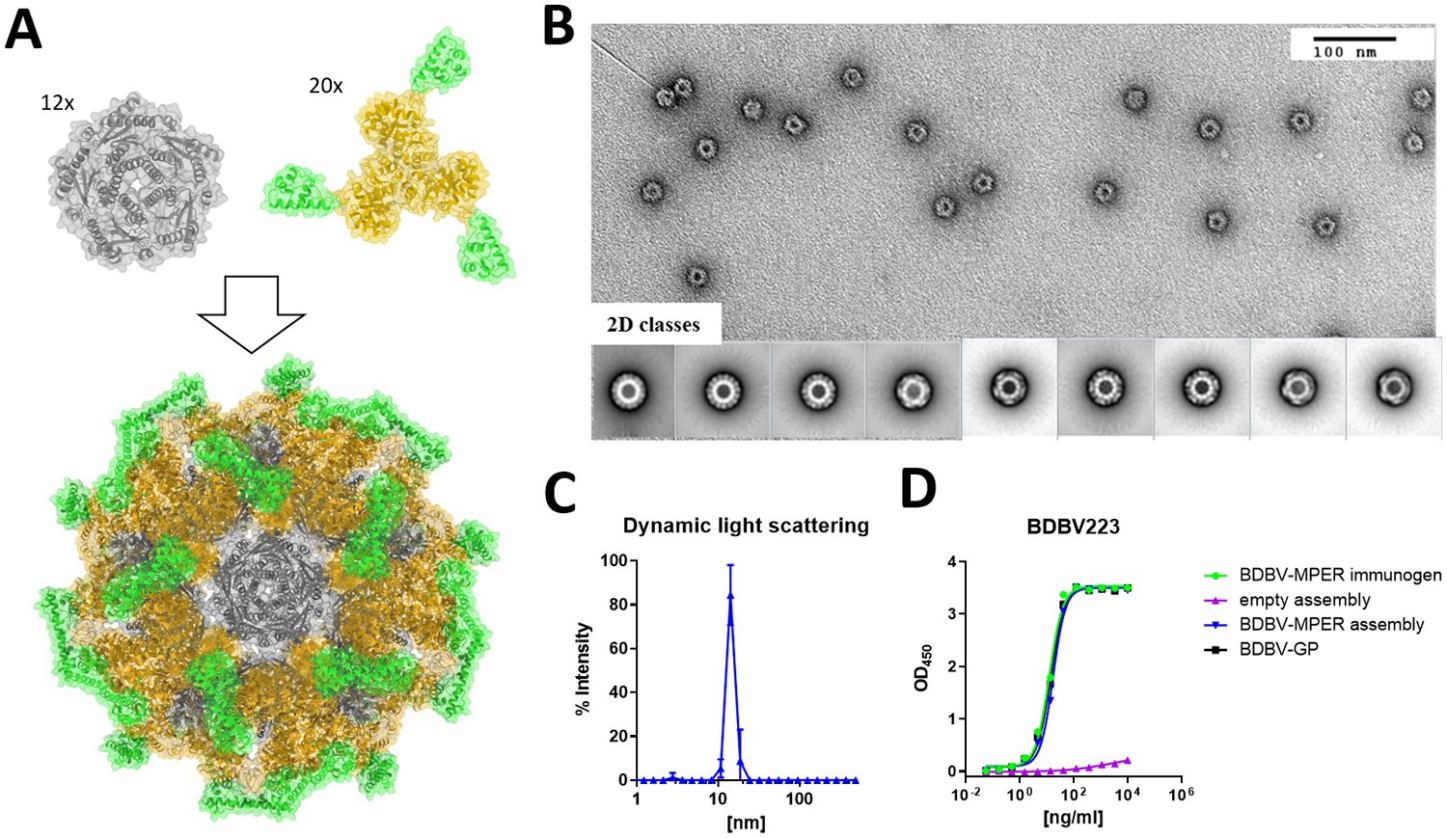
Design and characterization of self-assembling nanoparticles displaying the BDBV-MPER immunogen. (A) Model of the self-assembling nanoparticle displaying the BDBV-MPER immunogen on the trimeric component of the two-component I53-40 nanoparticle. (B) Negative stain electron microscopy 2D classes for I53-40 nanoparticles decorated with the BDBV-MPER immunogen. (C) Dynamic light scattering confirmed a homogeneous size distribution (n = 2, triplicates, exemplary experiment shown). (D) BDBV223 binding to the BDBV-MPER-bearing I53-40 nanoparticle observed by ELISA (n = 2, duplicates, SD). BDBV223 did not show any binding to empty I53-40 nanoparticles.

In an initial experiment, two New Zealand White (NZW) rabbits were injected with KLH-bound BDBV-MPER immunogen, and serum was collected after a prime immunization and three boosts to assess for antigen binding. Serum collected on day 90 after immunogen inoculation was tested for binding to ebolavirus GPs, and although serum binding to BDBV-GP was observed, neutralization activity against EBOV was not detected (S2 Fig). In order to achieve higher antibody titers, multivalent display of the immunogen on a self-assembling two-component nanoparticle platform was chosen [25, 27, 35]. Briefly, trimeric and pentameric scaffold proteins are mixed together to form regular icosahedral complexes comprising 60 copies of each subunit in the final complex [36]. Two-component nanoparticles have been used to multivalently display several class I fusion proteins through genetic fusion to the trimeric scaffold protein (*e*.*g*., the prefusion-stabilized RSV F protein [DS-Cav1] [25], the influenza hemagglutinin [28] or the HIV-envelope protein [26]). The BDBV-MPER immunogen is a small monomeric unit that we fused genetically to the trimeric components of three different nanoparticle scaffolds via a flexible linker (Fig 3). Trimeric proteins based on the reported designs of I53-40, I53_dn5, and I53-50 were tested for successful expression, assembly, and antibody binding. Additionally, two designs based on the assembly of a trimer and a dimer (termed I32-28 [36]) were tested. However, these did not express in the soluble fraction and were not considered for further studies. For all three successfully expressed nanoparticle component-immunogen fusions, surface exposure of the epitope was confirmed by ELISA binding to HR2-MPER antibodies (BDBV223, BDBV317, and BDBV340) (S3 Fig). For all three designs, the assembled nanoparticle was generated by mixing the trimeric component with the pentameric component and purified through size exclusion chromatography (SEC). Epitope exposure was confirmed by ELISA binding to BDBV223, BDBV317, and BDBV340 and off-targeting binding was probed using nanoparticles without surface decorated immunogen (S3 Fig). The I53-50 nanoparticle without immunogen displayed weak binding to BDBV223 in ELISA studies and was therefore excluded from further studies. Both I53_dn5 and I53-40 nanoparticles did not show any off-target antibody binding. I53-40-based nanoparticles were chosen for further studies based on a similar antibody binding profile to the BDBV-MPER immunogen (Figs 3D and S3). Structural integrity was verified using negative stain electron microscopy and dynamic light scattering. Both methods confirmed a homogenous composition of the BDBV-MPER immunogen-decorated nanoparticles (Fig 3B and 3C). However, representative negative stain EM 2D classes and a 3D reconstruction of the particle showed only the scaffold proteins since the immunogen could not be resolved, presumably due to flexibility in the linker between the displayed antigen and the nanoparticle scaffold (S4 Fig).

### Rabbit immunization with surface-displayed BDBV-MPER immunogen nanoparticles results in high antibody titers for Ebola GPs

Four antigens were selected for immunization studies: 1.) the BDBV-MPER immunogen, 2.) the BDBV-MPER-KPL immunogen and as control antigens: 3.) an immunogen void of any HR2-MPER epitope, but the native sequence of the SAM domain, and 4.) the HR2-BDBV-MPER peptide (called BDBV-MPER peptide), each of which were displayed on the surface of the nanoparticle. Each group consisted of four NZW rabbits with prime and boost immunizations on days 1, 14, 42 and 56. Antigens were administered in Complete Freund’s Adjuvant for prime immunization and Incomplete Freund’s Adjuvant for boost immunizations. Blood was drawn on days 0, 28, 56 and 70.

**Fig 4:**
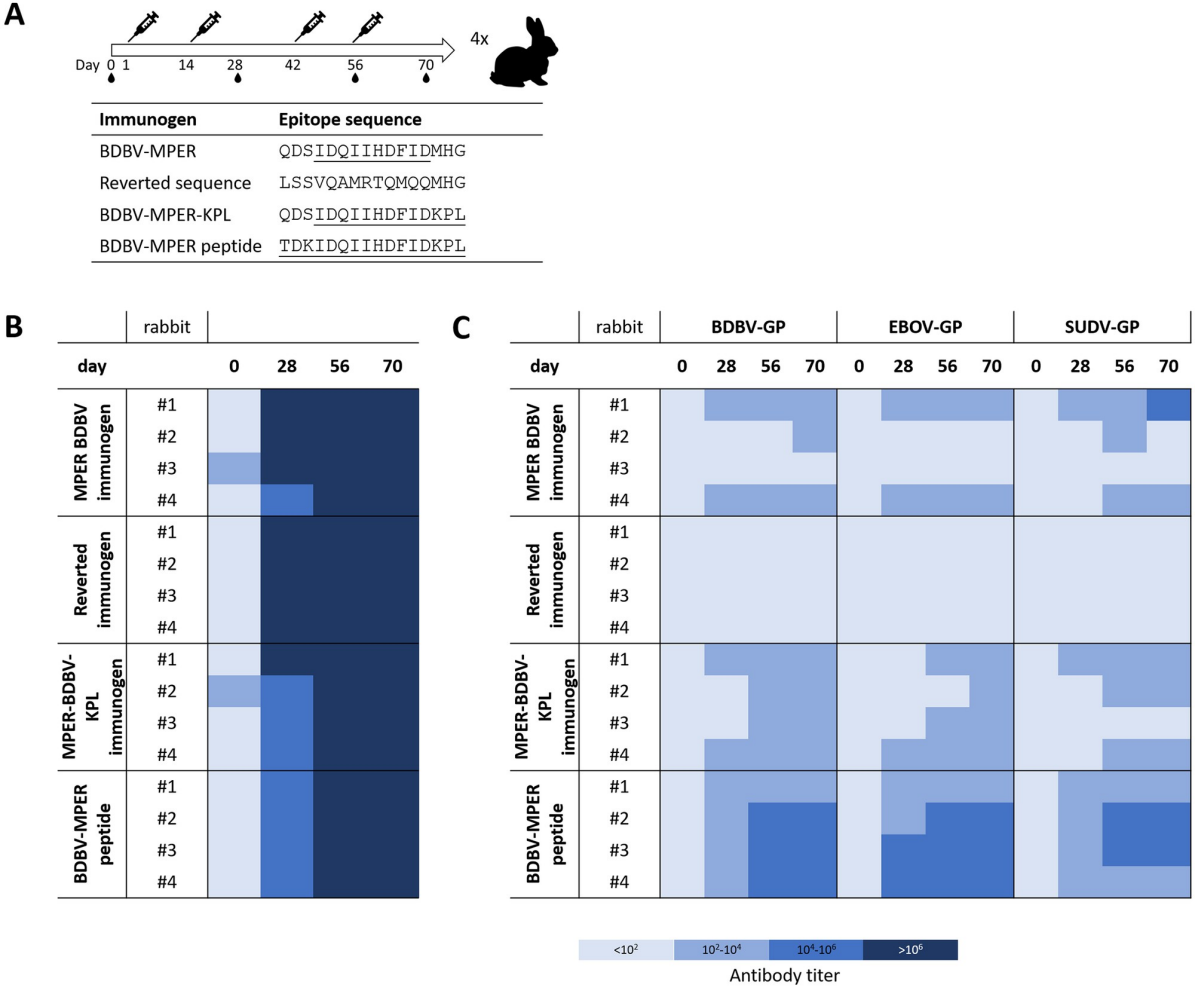
Serum binding from rabbits immunized with BDBV-MPER immunogens. (A) Immunization study set-up. Prime immunization, and three boosts were administered with 0.5 mg and 0.25 mg antigen, respectively. For prime immunization Complete Freund’s Adjuvant was used, while Incomplete Freund’s Adjuvant was used for boost immunizations. Blood was drawn on days 0, 28, 56, 70. (B) Serum binding titers to antigens used for immunization shows high titers for the nanoparticle formulation. (C) Serum binding titers for BDBV, EBOV, or SUDV GPs as determined by ELISA binding.

To assess immunogenicity, serum binding to the respective antigen was evaluated first. Strong serum binding was observed to the respective antigen for days 28, 56 and 70, while day 0 serum showed minimal or no binding (Figs 4B and S6). Next, serum binding to BDBV, EBOV, or SUDV GP was tested, and higher antibody titers were observed for rabbit sera immunized with nanoparticle-displayed immunogens, similar to the KLH-linked BDBV-MPER immunogen (S2, S7, S8 and S9 Figs). For the control group carrying the unmodified SAM domain scaffold protein, serum antibody binding was not detected at any time point to the three tested ebolavirus GPs. However, serum binding to the reverted scaffold antigen was strong. This finding confirms that serum antibody binding activity is mediated by the displayed epitope. In all three epitope-carrying groups, serum binding to BDBV GP was observed, except for rabbit #2 and #3 in the BDBV-MPER immunogen group, which showed very weak or no serum binding, despite having high antibody titers against the antigen (Figs 4B, S6, S7, S8 and S9). All other sera from the BDBV-MPER and BDBV-MPER-KPL groups contained antibodies that bound to BDBV and EBOV GP with up to a 1:100,000 dilution. Interestingly, serum binding was stronger for the BDBV-MPER peptide control group, with 3 out of 4 rabbit sera from day 70 binding to BDBV and EBOV GP with up to a 1:10,000,000 dilution. Cross-reactive binding to SUDV GP was observed for all three epitope carrying groups, with the BDBV-MPER peptide immunized group showing the highest antibody titers. Serum antibodies of rabbit #3 in the BDBV-MPER-KPL group did not bind to SUDV GP at all, despite having similar antibody titers for BDBV and EBOV GP as the other three animals in the group. Overall, antibody titers for SUDV GP seemed to be lower and delayed in response compared to BDBV or EBOV GP (Figs 4B, S6, S7, S8 and S9).

### Binding to MARV GP by one rabbit serum in the MPER-KPL group suggests cross-reactive immunization

**Fig 5:**
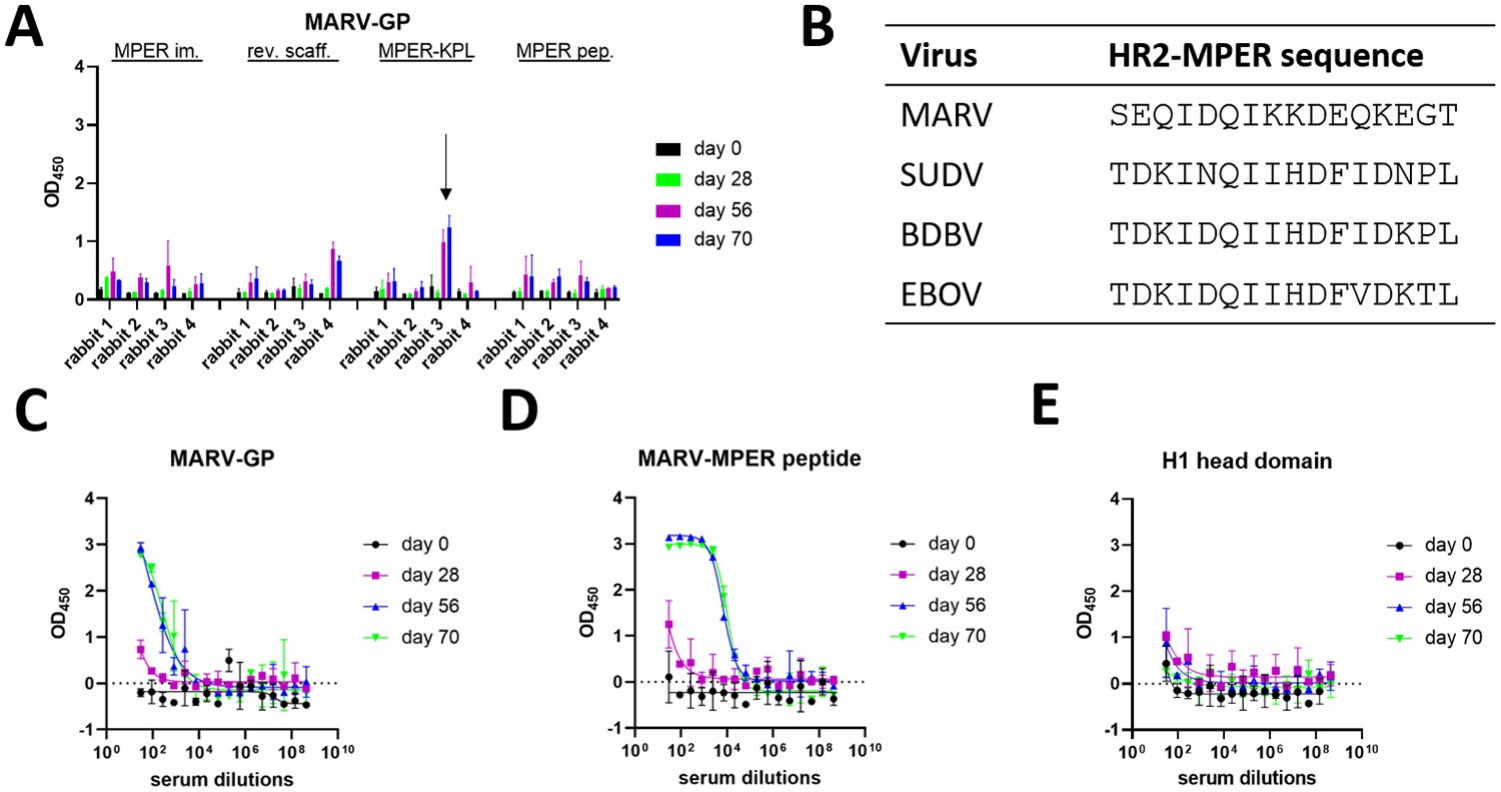
Serum from a MPER-KPL immunized rabbit shows cross-reactivity to MARV GP. (A) Screening of rabbit sera by ELISA at a dilution of 1:30. (B) Sequence alignment of HR2-MPER regions for the viruses BDBV, EBOV, SUDV, and MARV GPs. (C) Serum of BDBV-MPER-KPL immunized rabbit #3 bound to MARV GP in ELISA at days 56 and 70. (D) Serum of BDBV-MPER-KPL immunized rabbit #3 strongly bound to the MARV-MPER peptide (GIEDLSRNISEQIDQIKKDEQKEG) in ELISA. (E) As a control, an unrelated antigen, the monomeric hemagglutinin head domain for H1 (A/California/07/2009), was tested for serum binding to exclude off-target binding. (ELISA, n = 2, duplicates, exemplary experiment shown).

To test for cross-reactivity, rabbit sera were screened for binding to MARV GP at a dilution of 1:30 (Fig 5A). Initial binding was observed for serum from rabbit #3 of the BDBV-MPER-KPL immunized group. A possible MARV-HR2-MPER epitope has not been described in the literature. However, sequence alignment of all human pathogenic filoviruses displayed a hypothetical MARV-HR2-MPER epitope. This hypothetical epitope contains a number of different amino acids with some key residues sharing identity or amino acid characteristic similarities. For example, the following amino acids appear to be conserved: I623, D624, Q625, I626, D629, while other amino acids are exchanged for amino acids with similar properties, such as the exchange of D621 to E621 and H628 to K628. Interestingly, the MARV-HR2-MPER sequence is also preceded by a NxS sequence, which in all ebolaviruses is a NxT and carries a glycan as upper boundary of the epitope. It can therefore be rationalized that MARV-HR2-MPER might represent a possible epitope with similar properties to BDBV-HR2-MPER (Fig 5B).

Serum from rabbit #3 of the BDBV-MPER-KPL group was further analyzed for binding. At day 28, binding is barely observable at all, however, for day 56 and 70, binding is detectable for multiple dilutions, which suggests an induction of an antibody response. To test whether the observed binding is specific to the hypothetical MARV-HR2-MPER region, a peptide was used that contained the sequence GIEDLSRNISEQIDQIKKDEQKEG. Serum from days 56 and 70 strongly bound this peptide, whereas day 28 weakly bound. This suggests that cross-reactive antibodies to the hypothetical MARV-HR2-MPER region evolved gradually over time through somatic hypermutation. To confirm MARV-HR2-MPER region specific binding, serum was tested against an off-target antigen, hemagglutinin H1 head domain of A/California/07/2009, and failed to bind with specificity for any of the days tested. Interestingly, serum from rabbit #3 of the BDBV-MPER-KPL immunogen group bound to BDBV and EBOV GP only. These results suggest that immunization with an ebolavirus HR2-MPER epitope can elicit broadly-reactive polyclonal antibody responses, including MARV GP.

### Serum antibodies do not reveal neutralizing activity against BDBV or VSV/BDBV GP viruses

HR2-MPER antibodies, such as BDBV223, BDBV317 and BDBV340, potently neutralize authentic BDBV. However, these mAbs exhibit less neutralization activity and efficacy against EBOV and no observed activity against SUDV or Reston virus (RESTV) [14]. BDBV223 and BDBV317 protected mice inoculated with EBOV post-exposure but were far less effective in a guinea-pig challenge model [14]. When peptides derived from the HR2-MPER epitope were used to immunize rabbits, the serum antibodies displayed binding to BDBV, EBOV, and SUDV GP with varying levels of neutralization [14].

**Fig 6:**
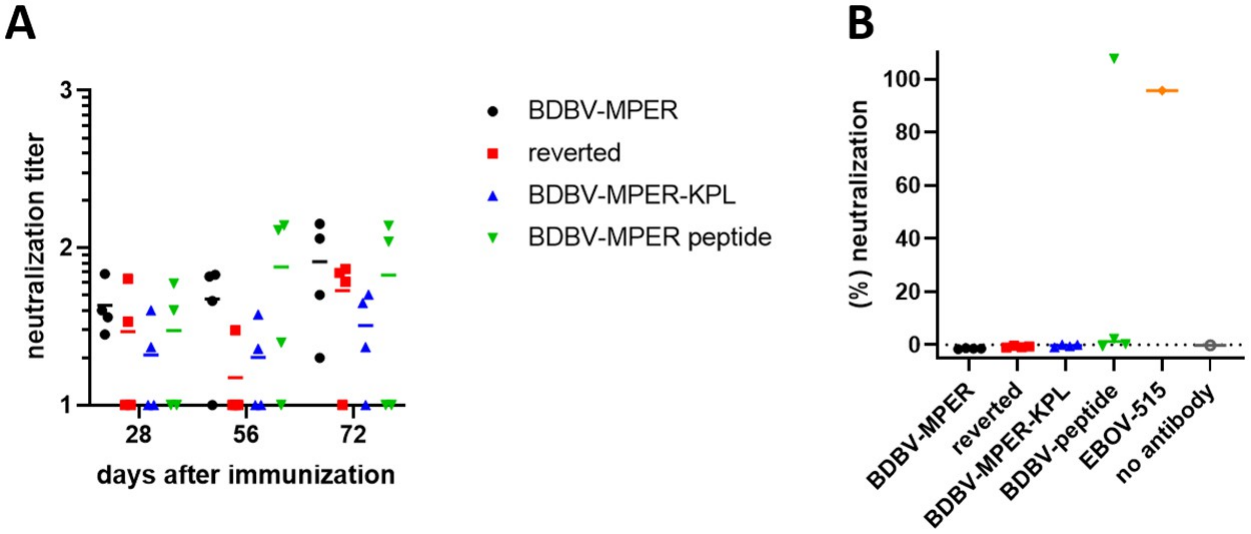
Neutralization studies from immunized rabbit sera. (A) Sera collected from individual immunized animals were tested for neutralization activity against BDBV, with no observable difference between the epitope carrying and non-carrying groups. (B) EBOV-515, a base-targeting antibody [8], neutralized chimeric infectious VSV/BDBV GP virus. Polyclonal antibodies from rabbits immunized on day 70 were not active, except for one sample from the BDBV-peptide immunization group.

Serum derived from rabbits immunized with BDBV-MPER immunogen, reverted immunogen, BDBV-MPER-KPL, or BDBV-MPER peptide were tested for neutralization activity. Pooled serum alone did not show a difference in neutralization activity for epitope-carrying immunogens versus reverted immunogen for BDBV (Fig 6A). There seems to be some unspecific reaction to BDBV, which can be observed by comparing the reverted scaffold group with the immunogen groups (Fig 6A). Subsequently, polyclonal antibodies (pAbs) were purified from rabbit serum using affinity chromatography, and pAb reactivity was verified by ELISA (S11 Fig). However, when testing pAbs neutralizing activity using recombinant chimeric vesicular stomatitis virus (VSV) expressing BDBV GP (VSV/BDBV GP), only one serum from the BDBV-MPER peptide group and the positive-control broadly-neutralizing GP base-specific antibody, EBOV-515 [8, 37] neutralized the virus (Fig 6B).

### NMR-derived structural models of the BDBV-MPER immunogen support the Rosetta-designed structure

**Fig 7:**
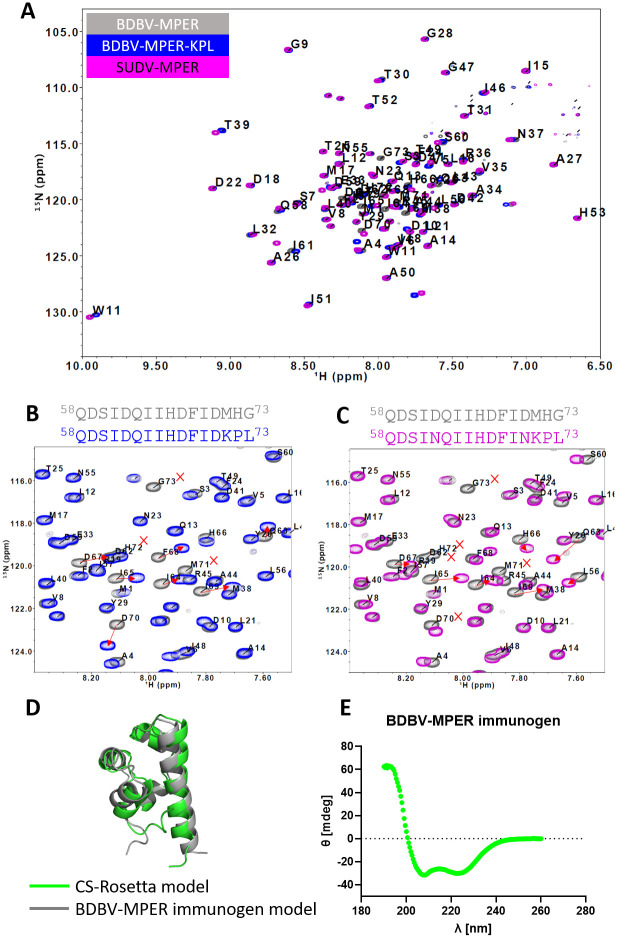
Structural validation of the BDBV-MPER immunogen design using solution NMR. (A) ^1^H-^15^N TROSY spectra of BDBV-MPER, BDBV-MPER-KPL, and SUDV-MPER immunogens. Backbone resonance assignments are indicated by one-letter amino acid code and sequence number. Resonances that are unassigned correspond to the purification tags. (B) Overlay of ^1^H-^15^N-TROSY spectra of BDBV-MPER and BDBV-MPER-KPL immunogens. The only change in the protein sequence is ^71^MHG^73^ to ^71^KPL^73^ meaning that there are no corresponding peaks for residues 71–73. Red arrows indicate shifted peaks. (C) Close-up view of the BDBV-MPER, BDBV-MPER-KPL and SUDV-MPER immunogen ^1^H-^15^N-TROSY spectra with the BDBV-MPER backbone assignment. (D) The NMR-derived model of BDBV-MPER in green is overlaid with the Rosetta-designed BDBV-MPER immunogen in grey. The backbone RMSD between the experimental and predicted structure was 2.4 Å. (E) CD spectrum of the BDBV-MPER immunogen.

We structurally validated the BDBV-MPER immunogen design using solution NMR spectroscopy. ^15^N and ^13^C isotopically-labeled protein was cultured in minimal medium and expressed in *E*. *coli* for purification and NMR measurements. The backbone amide ^1^H, ^15^N, and ^13^C resonances for the full-length BDBV-MPER immunogen were assigned using standard 3D NMR methods and covered all protein residues in the amino acid sequence, with the exception of residues belonging to the purification tag (Fig 7A). Assigned chemical shifts for all the BDBV-MPER immunogen nuclei have been deposited to the biological magnetic resonance bank (BMRB ID: 51377). We also ^15^N-labeled both the BDBV-MPER-KPL and SUDV-MPER immunogen to compare differences in the backbone chemical shifts with the BDBV-MPER immunogen using ^1^H-^15^N BEST-TROSY experiments [38, 39]. Overall, the chemical shifts for identical ^1^H-^15^N residue pairs for all three constructs were comparable, indicating that the structural configuration was not significantly perturbed. As expected, chemical shifts associated with the regions that differ in amino acid sequence changed, indicating an altered local chemical environment compared to the BDBV-MPER immunogen (Fig 7A).

For the BDBV-MPER-KPL immunogen that differs by three amino acids (K71, P72, L73) at the C-terminus, we observed ^1^H-^15^N chemical shift changes (δ_CS_) for the non-proline residues K71 and L73 of 0.225 and 0.104 ppm, respectively (S12 Fig). Neighboring amino acids Q63 through L73, showed the largest changes in chemical shift along the amino acid sequence compared to the BDBV-MPER immunogen spectrum. (Figs 7B and S12). Chemical shift changes for residues D67 and F68 were ambiguous and could not be confidently associated with a corresponding resonance in the BDBV-MPER immunogen spectrum. Overall, the chemical shift changes are small suggesting that the BDBV-MPER-KPL immunogen is in a similar confirmation to the BDBV-MPER immunogen.

The SUDV-MPER immunogen also has the K71, P72, and L73 exchange of amino acids at the C-terminus plus the additional exchange of residues with asparagine at positions N62 and N70. The SUDV-MPER spectrum overlayed with the BDBV-MPER spectrum shows a peak disappearance for residue and residue D70 is shifted, but in close proximity. We recognize here that this could be a new peak resonance from one of the mutated residues other than the new N62/N70 residue (Fig 7C). Similarly to the spectra for BDBV-MPER-KPL, all signals ranging from I61 to L73 showed the most pronounced changes in chemical shifts compared to the BDBV-MPER immunogen (S12 Fig). Interestingly, for residue I61, the I61 resonance for BDBV-MPER and BDBV-MPER-KPL are still overlapping (δ_CS_ = 0.048 ppm), but I61 from the SUDV-MPER immunogen construct is shifted more strongly (δ_CS_ = 0.147 ppm) (S13 Fig). Overall, the chemical shift changes are small and the largest changes are observed for the C-terminus residues ranging from I61—L73, suggesting that the new amino acids perturb that local chemical enviornment but do not significantly perturb the designed structure.

The assigned NMR chemical shifts for the BDBV-MPER immunogen residue sequence were used to predict the phi and psi backbone torsion angles using the software TALOS+ [40]. The predicted backbone phi and psi angles support the secondary structure of the Rosetta-designed BDBV-MPER immunogen. Interestingly, the C-terminal residues in the sequence, although forming a helix in our model, were predicted as loops by TALOS+ (S14 Fig). All assigned backbone and side chain NMR chemical shifts were used for structure prediction with CS-Rosetta [41–43]. The resulting highest-scoring model was compared to the design of BDBV-MPER immunogen with a calculated backbone RMSD of 2.4 Å (Fig 7D). We also analyzed the secondary structure using circular dichroism (CD) spectroscopy. The CD spectrum was recorded over a wavelength range of 190 nm to 260 nm, which resulted in a peak minimum at 222 nm and a peak maximum at 190 nm and indicates that alpha-helices dominate the secondary structure composition of the protein [44] (Fig 7E). Both the solution NMR and CD measurements indicate that the BDBV-MPER immunogen secondary structure agrees with our designed model.

## Discussion

In order to expand on the studies of immune responses from linear HR2-MPER peptide immunogens [14], we used computational immunogen design to stabilize the HR2-MPER epitope in the context of a protein backbone to achieve high antibody titers and investigate its ability to induce cross-reactive antibody responses in rabbits.

### Sidechain grafting is a preferred grafting method for clearly defined secondary structures

The applied computational protocols yielded one protein design that successfully expressed in *E*. *coli* and bound to BDBV223. This design, called BDBV-MPER immunogen, is based on a SAM domain (PDB:1B0X [31]) from the murine Eph receptor tyrosine kinase [31], in which the C-terminal residues were exchanged for the BDBV-HR2-MPER epitope though a sidechain graft and the dimeric interface was disrupted to allow formation of monomers. Although other computational grafting protocols were used to transplant the BDBV-HR2-MPER epitopes onto scaffold proteins, sidechain grafts were primarily selected for experimental testing, which does not enable evaluations of the success likelihood of one protocol over the other. It might be advantageous to choose a diverse set of designs generated from different methods, especially when selecting small numbers of designs for experimental testing. Many scaffold proteins with the desired topology are available for the linear and helical HR2-MPER epitope, which makes sidechain grafting a preferred method for epitope grafting. Sidechain grafting primarily relies on a backbone alignment between epitope and scaffold, which allows for the highest chances to recover the necessary backbone geometry of the epitope [15]. Therefore, it could be expected that the designed construct was derived through sidechain grafting. The co-crystal structure of BDBV-HR2-MPER peptide and BDBV223, however, contains some limitations: the BDBV-HR2-MPER peptide is strictly helical in the N-terminal half, which resulted in preference of the N-terminal part of the epitope during grafting. In computational design protocols, a high-resolution structure is advantageous because it ensures high confidence in the placement of each sidechain atom. The co-crystal structure, however, has an overall resolution of 3.6 Å, which brings about some uncertainty during design.

### Stronger binding of antibodies to BDBV-MPER immunogens compared to peptides

When testing the binding of the BDBV-MPER designs by the three known BDBV-HR2-MPER antibodies, BDBV223, BDBV317 and BDBV340, each antibody bound with EC_50_ values in the low ng/mL range. BDBV223, BDBV317 and BDBV340 were originally identified through binding to BDBV GP and epitope mapped through binding to peptides covering the BDBV-HR2-MPER amino acid sequence [14]. Naturally, peptides are not detected well in ELISA by antibodies and the difference in EC_50_ values between the BDBV-MPER immunogens and the HR2-MPER peptides may be derived from differences in sensitivity between these antigens. When probing the BDBV-MPER immunogen sequence with mutations investigated previously with HR2-MPER peptides, antibody binding remained strong as compared to the peptides. This observation might be due to the ability of the immunogen to maintain its backbone conformation in contrast to peptides.

### Immunogen flexibility on the surface of self-assembling two component nanoparticles

Since KLH did not result in very strong antibody titers during initial testing (S2 Fig), we employed a recently reported self-assembling nanoparticle platform [25, 36]. The nanoparticles were stable and homogenous as observed by various methods (EM, DLS, SEC). In negative stain EM, the immunogen was not resolved due to the flexible linker. Incubation with BDBV223 Fab resulted in loss of symmetry for the particle, indicating that a full homogenous coverage of the nanoparticle could not be reached under the incubation conditions (S15 Fig). This observation was also true in any crystallographic experiments–when crystallizing the trimeric scaffold protein that carried the immunogen, only the scaffold protein could be resolved (S16 Fig). It is most likely that the flexible linker prevented the formation of a regular crystal lattice.

### Rabbit immunization with nanoparticles resulted in high antibody binding titers

Four animal test groups were formed for immunization with the BDBV-MPER and BDBV-MPER-KPL immunogens, the BDBV-MPER peptide, and a native sequence scaffold immunogen. The antibody titers against all antigens were very strong starting at the first timepoint (day 28), which is in accordance with previously published data on nanoparticle display systems [25–27] (Fig 4B). When we assessed the serum binding against ebolavirus GPs, the response was stronger for days 56 and 70 of the BDBV-MPER-KPL and BDBV-MPER-peptide groups, which suggests that antibodies underwent somatic hypermutation to bind more strongly to the antigens. Substantial breadth was observed for BDBV223, but less so for BDBV317 and BDBV340, which only covered BDBV and EBOV GP. We next assessed serum binding to all three human pathogenic ebolavirus GPs. In the BDBV-MPER immunized group, serum from one animal did not bind any of the ebolavirus GPs tested, although it displayed binding to homologous antigen. In addition, serum from a second animal bound to the GPs relatively weak. There is currently no explanation as to why these two sera weakly reacted with the ebolavirus GPs. For serum from the other two animals, breadth of binding for EBOV and SUDV was observed, although the binding was much lower for SUDV. For the BDBV-MPER-KPL immunized group, sera from three animals displayed similar behavior, but the serum from one animal (rabbit #3) did not bind to SUDV GP at all. Overall, it can be noted that sera from the BDBV-MPER-KPL group developed antibodies more reliably against BDBV and EBOV. The BDBV-MPER peptide group had the highest antibody binding titers for all three ebolavirus GPs, and all four sera performed equally well.

A reason for this observed behavior could have resulted from the nanoparticle display system used in this study. The BDBV-MPER peptide was fused genetically to trimeric nanoparticles via its C terminus, tightly to the surface of the particle. The designed immunogen, although being a very small protein (72 aa), has been similarly fused via a flexible linker. The protein itself might occlude epitopes on the surface through interactions with each other or the surface of the nanoparticle and through motion. As the protein was not resolved on the surface of the nanoparticle in negative stain EM experiments, this suggests it behaves flexibly. Therefore, not as many epitopes might be exposed on the nanoparticle surface, resulting in insufficient cross-linking of B cell receptors.

### Cross-reactive binding to MARV GP

Immunized rabbit serum was also screened for MARV-GP binding at a single concentration from the four time points, although it is very rare that antibodies are cross-reactive for MARV and ebolavirus GPs [2]. However, serum from one animal displayed substantial binding at days 56 and 70 for MARV-GP. To confirm the specificity of this interaction, the serum was tested for binding to an off-target protein, the influenza H1 head domain, which failed to result in any measurable binding (Fig 4E). Serum antibody titers against MARV GP and a potential MARV-MPER 24-amino-acid peptide synthesized based on the alignment of the MARV GP sequence to EBOV GPs was determined. Interestingly, serum from rabbit #3 of the BDBV-MPER-KPL immunized group, does not bind to SUDV GP. An explanation for the observation could be that the antibody lineage raised in this particular animal differs from those observed in the other animals. The same could be true for mutations sampled during the process of somatic hypermutation. It was recently reported for the VRC034 antibody lineage that a very rare early mutation during somatic hypermutation can shape the immune response very dramatically [45]. A similar instance may occur here, especially since the breadth to the MARV-MPER develops only after a certain time and is only visible in serum collected at days 56 and 70 post-immunization. However, antibodies from humans targeting a hypothetical MARV-MPER have not been identified yet. As the Marburg virus glycoprotein adopts a different orientation between mucin-like domain to the core of the GP [46, 47] it might be more occluded as the ebolavirus HR2-MPER epitope. Extensive studies including the characterization of the human immune response to MARV GP are needed to further validate MARV-MPER as possible epitope.

### Lack of neutralizing activity

Serum from immunized rabbits was tested for neutralizing activity against authentic or chimeric VSV viruses. Previously, it was shown that BDBV223, BDBV317 and BDBV340 neutralize BDBV, but BDBV340 exhibits significantly less potency. In addition, BDBV223 and BDBV317 neutralized EBOV, but not SUDV. Lastly, BDBV223 and BDBV317 protected mice from EBOV challenge with 100% survival, however, these antibodies were less effective in a guinea pig model [14]. In the study by Flyak et al. [14] were peptide immunogens able to elicit neutralizing activity but with low breadth and largely with very moderate neutralization potency. The serum antibodies from the immunogen groups tested in this study did not neutralize BDBV in comparison to serum from the control group, and did not neutralize when IgG was purified from serum and tested against a VSV/BDBV GP virus (Fig 5). Only a single IgG fraction from one rabbit out of the BDBV-MPER peptide immunization group displayed any neutralizing activity against VSV/BDBV GP virus. However, the comparison to the study by Flyak et al. [14] is difficult as also a control group such as reverted scaffold was not available for comparison in the study. The lack of neutralization likely can be attributed to the fact that the designed proteins rely on a structure from a peptide-antibody complex that may not fully recapitulate the HR2-MPER epitope confirmation in the context of the GP trimer and viral membrane as it occurs during infection. This is supported by previous studies using epitope-focused immunogen design for RSV epitopes, in which the structural context of the epitope is important to successfully elicit the target antibody class [29].

### The structural conformation of HR2-MPER remains unresolved and more structural studies are needed to inform rational vaccine design

We used two biophysical tools, CD and solution NMR, to evaluate the overall structural configuration of the designed immunogen. The NMR backbone assignment for the BDBV-MPER immunogen allowed us to compare the relative structural similarity between the wild-type and mutant protein sequences. The mutant BDBV-MPER-KPL construct was ^15^N-labeled and the 1H-^15^N-TROSY spectra was superimposed with the BDBV-MPER 1H-^15^N-TROSY spectra for comparison. The chemical shifts remained at similar ppm values, indicating that the mutant constructs did not globally change the protein structure relative to the BDBV-MPER immunogen. As expected, we do observe chemical shift changes for residues in close proximity to the exchanged residues, and overall, these changes are more pronounced for residues 161 through L73. The BDBV-MPER spectrum compared to the SUDV-MPER immunogen shows a similar pattern for changes in chemical shift. We speculate that the epitope region is more flexible than our computational predictions suggest, which is supported by the decrease in the NMR order parameter (S2) between residues that reside in the alpha-helical regions (S2 = 0.9) vs. the last three amino acids (S2 = 0.6) in our TALOS prediction (S13 Fig). Since BDBV-MPER-KPL was mostly investigated to account for the last three residues in the HR2-MPER epitope, it can be concluded that this region is more flexible than expected. Although epitope-focused immunogen design in Rosetta has resulted in a collection of protocols for different cases, structural features that diverge from defined secondary structure are still challenging to capture and the availability of scaffold proteins is sparse. Further improvements in protein design will be necessary to tackle these design tasks.

**Fig 8:**
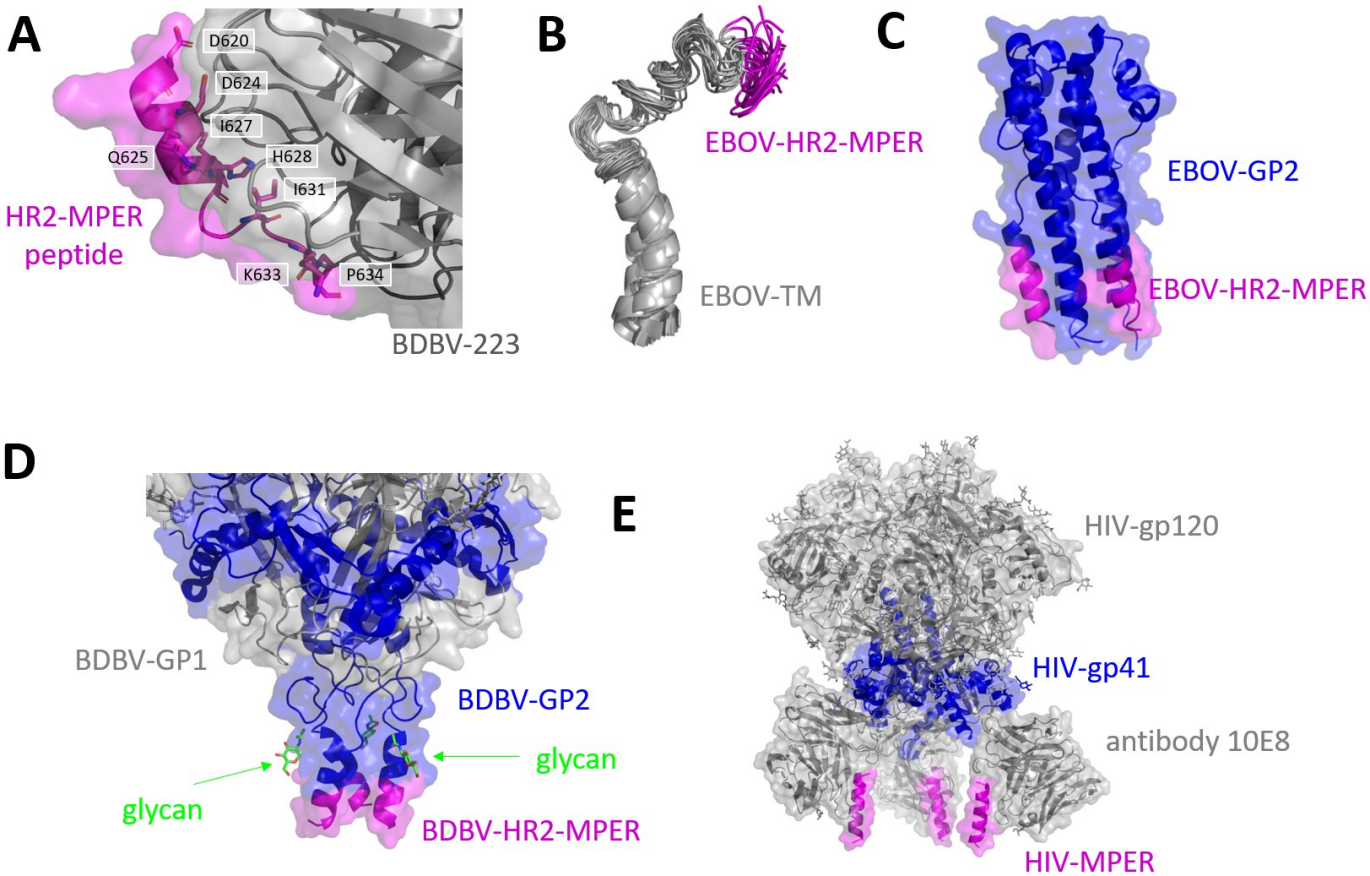
(A) Close-up of the interactions of BDBV-GP with the BDBV-MPER peptide via critical residues, PDB: 6N7J,—the only bound form of the EBOV MPER epitope [21]. (B) NMR solution structure of EBOV TM (PDB: 5T42), 20 lowest energy structures from DPC micelles at pH 5.5 [48]. (C) Post-fusion conformation of the EBOV-GP2 subunit (PDB: 2EBO). [52] (D) Close-up of a BDBV-GP structure (PDB: 6DZM) [32] with indicated glycosylation sites above the HR2-MPER epitope. (E) HIV envelope glycoprotein in a nanodisc in complex with 10E8, a HIV-MPER antibody, reconstructed from EM densities at a resolution of 5Å (PDB: 6VPX) [23].

The HR2-MPER region is notoriously hard to study using structural techniques. In BDBV-GP structures the stalk is never fully resolved (Figs 1A and 8D) [32, 37]. Additionally, the HR2-MPER epitope is covered by a possible glycan at its N-terminus (Fig 8D) and is situated close to the viral membrane, such that antibody binding cannot occur sterically in an upright position of the BDBV-GP [21]. It has therefore been postulated that ebolavirus GPs have intrinsic flexibility in their stalk region to enable recognition by antibodies [21]. Investigation of the EBOV transmembrane, MPER, and fusion loop regions using biomolecular NMR study with lipid mimetics, such as DPC micelles, also reported a substantial amount of flexibility in these regions (Fig 8B) [48]. The only other occurrence of the HR2-MPER epitope was observed in a crystal structure of the EBOV GP2 subunit in the post-fusion state. This state is not regarded as a good target for vaccine design as epitopes in post-fusion states are less likely to raise protective antibody responses and stabilization to the prefusion state is a vaccine design approach for many class I fusion proteins [49, 50].

MPER as an epitope for broadly neutralizing antibodies has also been described for other class I fusion proteins, primarily for HIV [51]. For the HIV envelope glycoprotein, similar behaviors have been postulated since the epitope is similarly enigmatic to the ebolavirus HR2-MPER due to flexibility and membrane proximity. A recent study reported the cryo-EM structure of HIV envelope glycoprotein in nanodiscs with the MPER antibody, 10E8, bound (PDB: 6VPX and Fig 8E), revealing hallmarks of HIV MPER antibody binding interface [23].

In the case of ebolaviruses, based on the observed BDBV223-HR2-MPER peptide co-crystal structure, flexibility has been postulated [21]. However, the lack of structural knowledge for the ebolavirus HR2-MPER epitope significantly impairs rational vaccine design efforts. Further structural investigation of the HR2-MPER epitope in ebolavirus GPs might shed light on the question of which structural conformation is important for the elicitation of neutralizing antibodies, such as BDBV223.

Another factor not captured in the BDBV-MPER immunogen design presented here, is the trimeric state of BDBV GP. As this design was solely based on the co-crystal structure of BDBV223 with the BDBV-peptide, it does not represent a trimeric state. However, it can be envisioned that BDBV223 contacts the other two domains and is dependent on the flexibility of the HR2-MPER epitope in the trimeric complex for binding.

As these structures are inconclusive for the exact conformation of BDBV-MPER needed as an epitope-focused immunogen, rational design of HR2-MPER-based immunogens remains challenging. Here, we demonstrate that a small helical protein carrying the epitope sequence at its C-terminus can elicit an antibody response. However, this epitope might not contain a conformationally correct epitope for elicitation of neutralizing antibody titers in rabbits. The elicitation of MARV-GP binding antibodies, however, allows to hypothesize that HR2-MPER is an interesting target for possible pan-filovirus targeting vaccines. As more structural investigations shed light on the conformational basis of neutralization by HR2-MPER antibodies, rational design efforts might be able to convey these designs into functional vaccine candidates.

## Conclusion

The foundation for pan-ebolavirus vaccines is the specific elicitation of antibodies with breadth against all three human pathogenic ebolaviruses, EBOV, BDBV and SUDV. In this study, the HR2-MPER epitope of BDBV was transferred using computational protein design in Rosetta onto a small scaffold protein to establish an epitope-specific immunogen. Using a self-assembling two-component nanoparticle system, the immunogen was prepared for immunization studies in rabbits and induced high titers of antibodies binding to BDBV and EBOV, and less pronounced to SUDV, which agrees with previous data for the HR2-MPER antibodies. One rabbit serum displayed binding to a potential HR2-MPER epitope on MARV, indicating that even pan-filovirus binding is possible based on the HR2-MPER epitope. However, the immunogens were not able to raise any neutralization activity in any test system employed in this study. Although all structural studies for the immunogen indicate that it adopts the modeled α-helical conformation, the HR2-MPER epitope observed in the co-crystal structure of BDBV223 with a 16-amino-acid peptide, might not fully explain the complex environment needed to raise neutralizing antibodies through structure-based vaccine design. This is the first attempt for epitope-focused immunogen design for ebolavirus epitopes. Although neutralization could not be reached, this study shows the potential for raising cross-reactive antibodies for *Filoviruses*. Further structural studies are warranted to guide efforts in epitope-focused immunogen design for *Filoviruses*.

## Methods

### Computational design of BDBV-MPER immunogens using Rosetta

The HR2-MPER epitope of BDBV223 was transplanted from the co-crystal structure of BDBV223 with a peptide containing the amino acids of HR2-MPER (PDB: 6N7J) [21] using three different grafting techniques in Rosetta. Small helical single chain proteins, that have been reported to be *E*. *coli* expressible, with a resolution <2.5 Å were collected from the PDB and were processed using RosettaRelax with backbone constraints. Subsequently, the following three grafting protocols were applied: sidechain grafting, backbone grafting and FoldFromLoops (1. Gen), as reported before [15–18, 33]. The resulting designs were scored for their total energy as a measure for stability and docked to BDBV223 to compare predicted binding energy as compared to the original peptide-antibody co-crystal structure. Furthermore, it was ensured that designs from all three protocols were selected for increasing diversity in the final ensemble. Designs based on the crystal structure 1B0X [31] were further modified to remove residues in the dimerization sites. A final selection of 11 designs was forwarded to experimental testing. Detailed instructions and sequences (S1 Text, and S1 and S2 Tables) are reported in the **SI**.

### Expression and purification of immunogen designs

Designs were codon-optimized in pET15b, containing a His-tag and a thrombin-cleavage site for expression in BL21 (DE3). They were transformed and transferred to Lysogeny Broth (LB) medium. When reaching an OD of > 0.8, isopropyl-β-D-1-thiogalactopyranoside (IPTG) was added to a final concentration of 50 μM. Cultures were incubated for 3 h at 37°C, centrifuged at 6,500 rpm at 4°C for 20 min in a Beckman Avanti JXN26 (Brea, CA, USA). The supernatant was discarded, and the pellet transferred and frozen at -80°C. The pellet was resuspended in buffer containing 25 mM Tris, 240 mM NaCl, 1 mM DTT, 20 mM imidazole, pH 8.0. The suspension was homogenized and subsequently sonicated at an amplitude of 60% for a total of 10 min. After centrifugation at 20,000 rpm at 4°C for 20 min was the supernatant transferred to a column containing Ni-NTA resin (Qiagen, Venlo, Netherlands). The resin was washed with one column volume and the protein eluted with buffer containing 25 mM Tris, 240 mM NaCl, 1 mM DTT and 400 mM imidazole. Protein solution was concentrated and underwent size exclusion chromatography (SEC) on a Superdex 75 (GE Healthcare, Chicago, Il, USA) in buffer containing 25 mM Tris, 150 mM NaCl and 1 mM DTT, pH 8.0. Fractions containing pure protein as controlled on SDS-PAGE were pooled and concentrated for further use.

### Site-directed mutagenesis

Based on the BDBV-MPER design, the corresponding SUDV- and EBOV-, as also a second generation BDBV-MPER design, namely BDBV-MPER-KPL, were introduced through site-directed mutagenesis using the QuickChange Lightning Kit (Agilent, Santa Clara, CA, USA) according to the instructions of the supplier. Sequences were controlled through Sanger sequencing.

### Cell lines

ExpiCHO (hamster, female origin) and FreeStyle 293F (human, female origin) cell lines were purchased from Thermo Fisher Scientific (Waltham, MA, USA) and cultured according to the manufacturer’s protocol.

### Antibody expression and purification

For recombinant monoclonal antibody (mAb) production, cDNA encoding the genes of heavy and light chains were cloned into DNA plasmid expression vectors encoding IgG (IgG1, IgG3 or IgG4)—or Fab- heavy chain [53] and transformed into *E*. *coli* cells. mAb proteins were produced following transiently transfection of FreeStyle 293F or ExpiCHO cells following the manufacturer’s protocol (Thermo Fisher Scientific, Waltham, MA, USA) and were purified as described above. mAbs were purified from filtered culture supernatants by fast protein liquid chromatography (FPLC) on an ÄKTA instrument (Cytiva, Chicago, Il, USA) using HiTrapMabSelect Sure or HiTrap Protein G columns (GE Healthcare, Chicago, Il, USA). Purified mAbs were buffer exchanged into phosphate-buffered saline (PBS), filtered using sterile 0.45-μm pore size filter devices (Merck Millipore, Burlington, MA, USA), concentrated, and stored in aliquots at -80°C until use.

### Expression and purification of Ebola glycoproteins

The ectodomains of EBOV GP_Δ_TM (residues 1–636; Makona variant; GenBank: KM233070), BDBV GP_Δ_TM (residues 1–643; strain200706291 Uganda; GenBank: NC_014373), SUDV GP_Δ_TM (residues 1–637; Gulu variant; GenBank: NC_006432), and MARV GP_Δ_TM (residues 1–648; Angola2005 variant; GenBank: DQ447653) were expressed transiently in Expi293F cells with a C-terminal strep II tag using the pcDNA3 plasmid vector. Secreted proteins were purified from filtered supernatant using 5 mL StrepTrap HP columns (Cytiva, Chicago, Il, USA) following the manufacturer’s protocol, and subjected to size exclusion chromatography using Superose 6 (Cytiva, Chicago, Il, USA) and buffer exchanged into PBS. Trimer fractions were pooled from SEC tested in ELISA through binding against control antibodies.

### Binding studies using ELISA

Wells of microtiter plates were coated with purified, recombinant EBOV, BDBV, SUDV, or MARV GP_Δ_TM at 1 μg/Land incubated at 4°C over-night. Protein antigens were coated at a concentration of 1 μg/ml in PBS, MARV-MPER-peptide was obtained from GenScript (Piscataway Township, NJ, USA) and coated at 4 μg/mL. Plates were blocked with 2% non-fat dry milk and 2% normal goat serum in DPBS containing 0.05% Tween-20 (DPBS-T) for 1 hr. The antibody or serum was diluted in in blocking buffer, added to the wells, and incubated for one hour at ambient temperature. The bound antibodies were detected using goat anti-human IgG conjugated with HRP (Southern Biotech, Birmingham, AL, USA) and TMB substrate (Thermo Fisher Scientific, Waltham, MA, USA) for human IgG or goat anti-rabbit IgG conjugated with HRP (Southern Biotech, Birmingham, AL, USA) for rabbit sera. Color development was monitored, 1 N hydrochloric acid was added to stop the reaction, and the absorbance was measured at 450 nm using a spectrophotometer (Biotec EL406, Biotec, Winooski, VT, USA). For dose-response and cross-reactivity assays, serial dilutions of plasma or purified mAbs were applied to the wells in triplicate or quadruplicate, as detailed above. EC_50_ values for mAb binding were determined using Prism 8.3 software (GraphPad, San Diego, CA, USA) after log transformation of antibody concentration using sigmoidal dose-response nonlinear regression analysis. Similarly, a non-linear regression analysis was performed on the resulting curves to calculate plasma dilution that yielded a half-maximum OD 450 nm value. Antibody titer in plasma was expressed as the inverse of plasma dilutions. Data analysis was performed using GraphPad Prism 8.3.

### Expression and purification of nanoparticle displayed BDBV-MPER immunogens

BL21(DE3) competent bacteria were transformed with pET11a vectors carrying the respective gene. Starting cultures were inoculated overnight in LB medium and transferred to fresh LB medium 18h later. Protein expression was induced with a final concentration of 5 μM of IPTG upon reaching an OD of > 0.8. Protein expression was abrogated after 3 h as medium was centrifuged at 6,500 rpm in an Avanti JXN26 (Beckman, Brea, CA, USA) at 4°C for 20 min. Subsequently, supernatant was decarded and pellet frozen at -80°C. Cells were reconstituted and homogenized in buffer containing 25 mM Tris, 240 mM NaCl, 1 mM DTT, 20 mM imidazole, pH 8.0. After sonication with an amplitude of 60% for in total 10 min, the suspension was centrifuged at 20,000 rpm for 20 min at 4°C. Supernatant was transferred to columns filled with Ni-NTA resin (Qiagen, Venlo, Netherlands), washed with ten column volumes of buffer and eluted with buffer containing 25 mM Tris, 240 mM NaCl, 1 mM DTT, 400 mM imidazole, pH 8.0. Protein solution was concentrated using Amicon filter tubes (Merck KGaA, Darmstadt, Germany), applied to a Superdex 200 (GE Healthcare, Chicago, Il, USA) column and eluted using buffer 25 mM Tris, 150 mM NaCl, 1 mM DTT, pH 8. Fractions showing a single band of the expected size were pooled and concentrated. Trimeric component of the particle was incubated for 1 h and room temperature or 24 h at 4°C in an equimolar ratio with the pentameric component of the nanoparticle on a rocking shaker. Pentameric component of the self-assembling nanoparticle was purified as described before [25]. Subsequently, the mixture was applied to a Superose 6 Increase column (GE Healthcare, Chicago, Il, USA). Nanoparticle eluted in the void volume, was combined and concentrated.

### Size determination of self-assembling nanoparticle

Particle size in solution were determined using dynamic light scattering with the Wyatt DynaPro NanoStar (Wyatt, Santa Barbara, CA, USA). The protein solution was titrated to a concentration of roughly 1 mg/mL, filtered, transferred into a NanoStar Disposable MicroCuvettes (Wyatt, Santa Barbara, CA, USA) and compared to buffer scans. Only when buffer scans showed no signal, 10 scans were recorded in triplicate at 25°C after a resting period of 5 min.

### Negative stain electron microscopy of self-assembling nanoparticle

#### Negative stain grid preparation

For screening and imaging of negatively stained (NS), ~3 μL of I53-40 sample at concentrations of ~15 μg/mL were applied to continuous carbon film on 400 mesh copper EM grids that was glow discharged (Electron Microscopy Sciences, Hatfield, PA, USA). The grids were stained with 0.75% Uranyl formate (UF).

#### Screening, data collection, and image processing

NS grids were screened on a FEI Morgagni (ThermoFisher, Waltham, MA, USA) microscope operating at 100 kV with AMT 1k × 1k CCD camera to verify sample and grid quality. Data collection from NS grids were recorded on FEI TF20 (Thermo Fisher, Waltham, MA, USA) operate at 200 kV with US4000 4k × 4k CCD camera (Gatan, Pleasanton, CA, USA) and controlled by SerialEM [54]. The data set was collected at mag of 50 K with Å/pix of 2.18 with defocus range of 1.4 to 1.8 and a dose rate of ~30.0 e/A2. 120 micrographs were collected and process in Scipion [55]. The data set was binned to 4.36 Å/pix and 3,357 particles was autopicked and extract at a box size of 128 pixel. Multiple rounds of 2D classification [55–57] were performed to clean the dataset and separate the different species into distinct classes.

### Endotoxin removal for rabbit immunization

Endotoxin removal was performed using Pierce High Capacity Endotoxin Removal Spin Columns (Thermo Fisher Scientific, Waltham, MA, USA) of varying sizes according to the instruction of the suppliers. Endotoxin concentrations were tested with an Endosafe nexgen-MCS spectrophotometer (Charles River Laboratories, Wilmington, MA, USA) using Limulus Amebocyte Lysate (LAL) Cartridges (Charles River Laboratories, Wilmington, MA, USA). Sample solutions were prepared by diluting 1:100 using endotoxin-free water (Charles River Labratories, Wilmington, MA, USA) and diluted as needed to reach the sensitivity range of the cartridge (0.005 EU/mL to 10–0.1 EU/mL).

### Rabbit immunization

NZW rabbits that are Specific Pathogen-Free were immunized subcutaneously with 0.25 mg of the respective immunogen (Thermo Fisher, Custom rabbit polyclonal antibody production) at for four different sites in Complete Freund’s Adjuvant. Subsequently, the animals were boosted three times subcutaneously with the same conjugates in Freund’s incomplete adjuvant at days 14, 42 and 56. Crude serum and purified pAbs were tested in ELISA for binding to the respective immunogen and *Filovirus* GPs. The presence of antibodies bound to the antigen was determined using goat anti-rabbit IgG HRP conjugate (Southern Biotech, Birmingham, AL, USA).

### Purification of IgG from rabbit serum

4 mL of rabbit serum were diluted 1:5 with PBS and filtered using 0.2 μM filter units (Merck Millipore, Burlington, MA, USA). The solutions were consecutively applied to a ProteinMaker parallel purification system (ProteinBioSolutions, Gaithersburg, MD, USA) using 1 ml HiTrap MabSelect SuRe columns (Cytiva, Chicago, Il, USA) and washed with PBS. Polyclonal IgG were eluted using 0.2 M sodium acetate, pH 3.3buffer. Samples were neutralized using 1M Tris pH 8.0 buffer, filtered through a 0.2 μM filter and buffer-exchanged into PBS using Amicon Ultra-4 50 kDa Centrifugal Filter Units (Merck Millipore, Burlington, MA, USA). Final concentrations were measured using a NanoDrop spectrophotometer (ThermoFisherScientific, Waltham, MA, USA) and pAbs stored at 4°C until use.

### Virus neutralization assays

For chimeric VSV/BDBV GP neutralization assays, we used a high-throughput and quantitative real-time cell analysis assay (RTCA) and xCELLigence Analyzer (ACEA Biosciences Inc., San Diego, CA, USA) that assesses kinetic changes in cell physiology, including virus-induced cytopathic effect (CPE) [58]. 50 mL of cell culture medium (DMEM supplemented with 2% FBS) was added to each well of a 96-well E-plate to obtain background reading. Eighteen thousand (18,000) Vero-E6 cells in 50 mL of cell culture medium were seeded per each well and plate was placed on the analyzer. Measurements were taken automatically every 15 min and the sensograms were visualized using RTCA software version 2.1.0 (ACEA Biosciences Inc., San Diego, Ca, USA). rVSV/BDBV GP virus [59] (0.3 MOI, ~6,000 PFU per well) was mixed with eight two-fold dilutions of individual mAbs starting at 25mg/mL in a total volume of 100 mL and incubated for 1 hat 37°C. At 12 h after seeding the cells, the virus/mAb mixtures were added in two replicates to the cells in 96-well E-plates. Wells containing virus only in the absence of mAb and wells containing only Vero cells in medium were included on each plate as controls. Plates were measured continuously (every 15 min) for over 48 h to assess virus neutralization. Normalized cellular index (CI) values at the endpoint (42 h after incubation with the virus) were determined using the RTCA software version 2.1.0 (ACEA Biosciences Inc.). Results were expressed as percent neutralization in the presence of a particular mAb relative to no-CPE control wells minus CI values from control wells with maximum CPE.

Biosafety level 4 neutralization assays were performed using EBOV-eGFP virus or the chimeric EBOV-eGFP virus in which GP was replaced with its counterpart from BDBV (strain Uganda) or from SUDV (strain Gulu) as described previously [60]. The biological isolate BDBV strain Uganda was used in experiments displayed in panel Fig 6A. For those, rabbit serum samples were heat-inactivated for 30 min at 56°C and diluted in a 2-fold serial fashion in MEM (Gibco, Thermo Fisher Scientific, Waltham, MA, USA) with HEPES (Corning, Corning, NY, USA), gentamicin sulfate (Cellgro, Corning, NY, USA) and 10% guinea pig complement (MP Biomedicals, Irvine, CA, USA). 50 μL of each serum dilution was mixed with 200 PFU of virus in 50 μL. The serum/virus mixtures were incubated for 1 h at 37°C. Fifty μL of the serum/virus mixtures were then transferred to Vero E6 cell monolayers in flat-bottom 96-well plates and incubated for 1 h at 37°C. The serum/virus mixture was then removed and replaced with 1:1 overlay composed of 1% methylcellulose (Fisher Chemical, Waltham, MA, USA) and 2X MEM (Gibco, Thermo Fisher Scientific, Waltham, MA, USA) supplemented with gentamicin sulfate and 4% FBS (Gibco, Thermo Fisher Scientific, Waltham, MA, USA). Plates were incubated 5 days at 37°C, then fixed with 10% neutral buffered formalin (Fisherbrand, Thermo Fisher Scientific, Waltham, MA, USA) according to approved SOP and removed from biocontainment. Plates were washed 3 times with 1x DPBS (Corning, Corning, NY, USA) before a 1 h blocking step in 1x DPBS with 5% milk (blotto, Santa Cruz Biotechnologies, Dallas, TX, USA). BDBV plaques were immunostained with 1 μg/mL human monoclonal BDBV52 as primary antibody. Plates were washed three times in 1X DPBS before addition of HRP-conjugated goat anti-human IgG (KPL) at dilution 1:2,000 as secondary antibody. Both primary and secondary antibodies were diluted in blotto. Finally, plates were washed three times in 1x DPBS (Corning, Corning, NY, USA) and plaques were revealed by a 30 min incubation at 37°C with AEC substrate (enQuire Bioreagents, Littleton, CO, USA).

### Expression and purification of ^15^N- and ^15^N,^13^C-labeled BDBV-MPER immunogens

The BDBV-MPER, SUDV-MPER and EBOV-MPER immunogen design constructs were expressed and purified as described above with the following modifications for the preparation of NMR samples. Recombinant DNA was transformed in *E*. *coli* BL21(DE3) cells. Cells were grown overnight in 5 mL of LB-medium at 37°C, centrifuged at 4,000 rpm for 10 min and pellet subsequently transferred to 1L of M9 minimal medium containing ^15^N-labelled ammonium sulfate (^15^NH_4_)_2_SO_4_ (Cambridge Isotope Laboratory, Tewksbury, MA, USA) containing ^15^N-labelled ammonium sulfate (^15^NH_4_)_2_SO_4_ and 0.2% ^13^C-glucose (Cambridge Isotope Laboratory, Tewksbury, MA, USA). After culturing in M9 medium until cell density reached an OD of > 0.8, protein expression was induced by adding IPTG to a final concentration of 50 μM. Cells were harvested after additional incubation at 37°C overnight and harvested by centrifugation. For ^15^N,^13^C-labelled protein, the incubation was prolonged for another 6h. Uniformly ^15^N- and ^15^N,^13^C-labeled samples were exchanged into NMR buffer containing 50 mM imidazole, 50 mM NaCl, 0.2 mM EDTA, and 7% D_2_O (pH 6.5). The final concentration of the sample ranged from 0.38 mg/mL for ^15^N,^13^C-BDBV-MPER immunogen, to 2.7 mg/mL for ^15^N-MPER-SUDV. Protein purity was confirmed to be greater than 95% by SDS-PAGE. Both the ^15^N-labelled SUDV- and EBOV-immunogen precipitated after 72 h. The ^15^N,^13^C -labelled BDBV-immunogen was stable over many months.

### Backbone assignments and structure calculations for the BDBV-MPER immunogen

A suite of 2D/3D heteronuclear NMR spectra were recorded at 298K using a Bruker 800 MHz spectrometer equipped with a 5 mm proton optimized triple resonance “inverse”, z-axis gradient cryoprobe. Backbone amide resonances were assigned using 2D ^15^N-^1^H HSQC, 3D HNCA/HN(CO)CA, HNCO/HN(CA)CO, and 3D CBCANH/CBCA(CO)NH. All spectra were processed using the software TopSpin 3.6.2 (Bruker, Billerica, MA, USA) and analyzed using the program NMRViewJ [61, 62]. Assigned chemical shift lists from the 2D/3D NMR experiments were used as input to calculate the BDBV-immunogen structure in CS-Rosetta [42].

### Circular dichroism spectroscopy

Buffer of protein solution was exchanged for a 20 mM KH_2_PO_4_ buffer, pH7.4, filtrated and diluted to a final concentration of 0.18 mg/mL. Spectra were recorded on a Jasco 800 spectrometer (Jasco, Tokyo, Japan) at room temperature within a range of 260–190 nm with a data pitch all 4 s at steps of 0.5 nm. Data were processed using GraphPad Prism 8.3.

## Supporting information

S1 Table The sequences of the selected designs ordered based on scaffold protein. (DOCX)

S2 Table Sequences of immunogens carrying BDBV-MPER, BDBV-MPER-KPL, EBOV-MPER, SUDV-MPER, BDBV-MPER-I631V, BDBV-MPER-D624N and BDBV-MPER-KPL-D624N. (DOCX)

S1 Fig ELISA binding to 1b0x-2-based immunogens, carrying different variations in the epitope sequence (compare Fig 2 or S2 Table for sequences). (TIF)

S2 Fig Serum binding to soluble Ebola GPs for two rabbits immunized with keyhole-limpet hemocyanin (KLH)-coupled MPER-BDBV immunogen, or a control solution. (TIF)

S3 Fig Binding experiments using the three HR2-MPER antibodies BDBV223, BDBV317 and BDBV-320 for the three self-assembling nanoparticle designs I53-50, I53-40 and I53-dn5, which were tested with a linked immunogen, in its trimeric form with immunogen or fully-assembled with pentameric component. Nanoparticles displayed 60 copies of the BDBV-MPER immunogen on its surface.
(TIF)

S4 Fig 3D reconstruction of I53-50-BDBV-MPER immunogen from negative stain electron microscopy experiments as surface and mesh. Notably, the immunogen cannot be resolved.
(TIF)

S5 Fig Nanoparticle antigen for rabbit immunization was tested for antibody binding prior to immunization using BDBV223, BDBV317 and BDBV340. (TIF)

S6 Fig Serum binding to antigens in ELISA. **A.** Serum binding of rabbits immunized with nanoparticle displayed BDBV-MPER immunogen to BDBV-MPER immunogen on nanoparticles. **B.** Reverted immunogen. **C.** BDBV-MPER-KPL immunogen. **D.** BDBV-MPER peptide.
(TIF)

S7 Fig Serum binding to BDBV-GP for sera from all four blood draw time points. **A.** Serum binding of rabbits immunized with nanoparticle displayed BDBV-MPER immunogen to BDBV-MPER immunogen on nanoparticles. **B.** Reverted immunogen. **C.** BDBV-MPER-KPL immunogen. **D.** BDBV-MPER peptide.
(TIF)

S8 Fig Serum binding to EBOV-GP for sera from all four blood draw time points. **A.** Serum binding of rabbits immunized with nanoparticle displayed BDBV-MPER immunogen to BDBV-MPER immunogen on nanoparticles. **B.** Reverted immunogen. **C.** BDBV-MPER-KPL immunogen. **D.** BDBV-MPER peptide.
(TIF)

S9 Fig Serum binding to SUDV-GP for sera from all four blood draw time points. A. Serum binding of rabbits immunized with nanoparticle displayed BDBV-MPER immunogen to BDBV-MPER immunogen on nanoparticles. B. Reverted immunogen. C. BDBV-MPER-KPL immunogen. D. BDBV-MPER peptide.
(TIF)

S10 Fig Serum binding to MARV-GP for day 70 sera. As MARV-MPER peptide, a peptide with the following sequence was used: GIEDLSRNISEQIDQIKKDEQKEG.
(TIF)

S11 Fig Antigen binding of purified polyclonal Abs from day 70 rabbit sera in ELISA against BDBV-GP. (TIF)

S12 Fig Change in chemical shift plotted over the immunogen sequence. As a reference the BDBV-MPER immunogen was used. * indicates positions where a peak was not matched. **A.** Change in chemical shift plotted over the sequence of BDBV-MPER-KPL in comparison to the BDBV-MPER immunogen. B. Change in chemical shift plotted over the sequence of SUDV-MPER in comparison to the BDBV-MPER immunogen.
(TIF)

S13 Fig Chemical shifts for I61 in BDBV-MPER (gray), BDBV-MPER-KPL (blue) and SUDV-MPER (magenta) in a close-up of the overlay of ^1^H-^15^N-TROSY spectra. (TIF)

S14 Fig TALOS prediction from chemical shift data. **A.** Prediction of secondary structure elements. Green indicates helical parts, light blue indicates β-sheets. **B.** Sequence of construct used for solution NMR experiments. **C.** Predicted unstructured areas have a low Random Coil Index Order Parameter.
(TIF)

S15 Fig 2D-classes from I53-40-BDBV-MPER-immunogen nanoparticles, incubated with BDBV223 Fab. (TIF)

S16 Fig Crystallographic studies using I53-50 BDBV-MPER and SUDV-MPER immunogens on trimeric scaffold protein do not resolve the immunogen (for detailed methods see S2 Text). **A**. Crystals of I53-50 trimeric component with BDBV-MPER immunogen. Crystals are not formed homogenous. **B**. Unit cell of the density from I53-50 BDBV-MPER trimeric construct at a resolution of 2.20 Å. As search model the trimeric component as reported in PDB: 5IM5 was used. **C.** Unit cell of the density from I53-50 SUDV-MPER trimeric construct at a resolution of 2.48 Å.
(TIF)

S1 Text Computational protocol. (DOCX)

S2 Text Supplemental Methods. (DOCX)
