# Supplementary material for: Epitope-focused immunogen design based on the ebolavirus glycoprotein HR2-MPER region
Source: PLoS Pathog. 2022 May 18;18(5):e1010518. doi: 10.1371/journal.ppat.1010518 (PMC9170092; doi:10.1371/journal.ppat.1010518)
Supplement: S1 Table — (DOCX) [file ppat.1010518.s001.docx]

**S1 Table.** The sequences of the selected designs ordered based on scaffold protein.

| Based on 1b0x [1]: |
| --- |
| >1b0x_0001_0001 (side chain graft)  FSAVVSVGDWLQAIKMDRYKDNFTAAGYTTLEAVANMTLDDLARIGITAITHQNKILDSIDQMIHDMIDMHG |
| >1b0x_0001_0002 (side chain graft, abolish dimerization by re-designing Mets)  FSAVVSVGDWLQAIKMDRYKDNFTAAGYTTLEAVRNMTLDDLARIGITAITHQNKIQDSIDQIIHDFIDMHG |
| >1b0x_0001_0003 (backbone graft)  TKDKIDQSIHDHIDKMDRYKDNFTAAGYTKSEAVVHMSQDDLARIGITAITHQNKILSLVQAARTAMQNLHG |
| >ffl1b0x_0165 (FoldFromLoops)  TSNSGSTESWARKRGLSWSADLARKMGFNTTALFANGGQEELKKAGVYDSEKIQRETDAIDQAIHDMIDGSG |
|  |
| Based on 3o3k [2]: |
| >3o3k_0001_0001 (side chain graft)  ERIKLVFKEKALEILMTIYYESLGGNDVYIQYIASKVNSPHSYVWLIIKKFEEAKMVECELEGRTKIIRLTDDGIDQAIHDKIDIDIMEN |
| >3o3k_0001_0002 (backbone graft)  ERIKLLFKEKALEIAMTIAYELAGGNDVYIQYIASKVNSPHSYVWLIIKKFEEAKMVECELEGRTKIIRSTADSIDQIIHDFIDIDVMEN |
|  |
| Based on 4g3o [3]: |
| >4g3o_0001_0001 (side chain graft)  RGSENLYFQGDLIDQAIHDQIDFPQVPYHLVLQDLQLTRSAQITADNILEGRI |
| >4g3o_0001_0002 (side chain graft)  RGEENKDFIDQLIHDAIDIQEMFPQVPYHLVLQDLQLTRSVEITTDNILEGRI |
|  |
| Based on 4o7q [4]: |
| >4o7q_0001_0001 (side chain graft)  AMESKYKEILLLTGLDNITDEELDRFKGFLSDEFNIATGKLHTANRIQVATLMIQNAGAVSAVMKTARINAKLNYYDLIDQLIHDKIDVDKQYK |
| Based on 4o9f [5]: |
| >4o9f_0001_0001 (side chain graft)  MTVAEDKTFQYIRQHHSNFSRIHVQEITPYLSCLTDDDIDQLIHDYIDWGNQDTLLELFTSLRCRNGWVHSLIGALRACELSGLADEVARIYHS |

**References**

1. Stapleton D, Balan I, Pawson T, Sicheri F. The crystal structure of an Eph receptor SAM domain reveals a mechanism for modular dimerization. Nat Struct Biol. 1999; 6(1):44-9.

2. Zhu JY, Fu ZQ, Chen L, Xu H, Chrzas J, Rose J, et al. Structure of the Archaeoglobus fulgidus orphan ORF AF1382 determined by sulfur SAD from a moderately diffracting crystal. Acta Crystallogr D Biol Crystallogr. 2012; 68(Pt 9):1242-52.

3. Crystal structure of the CUE domain of the E3 ubiquitin ligase AMFR (gp78). 2012.

4. Lu A, Kabaleeswaran V, Fu T, Magupalli VG, Wu H. Crystal structure of the F27G AIM2 PYD mutant and similarities of its self-association to DED/DED interactions. J Mol Biol. 2014; 426(7):1420-7.

5. Xu H, He X, Zheng H, Huang LJ, Hou F, Yu Z, et al. Structural basis for the prion-like MAVS filaments in antiviral innate immunity. Elife. 2014; 3:e01489.
