# Supplementary material for: Epitope-focused immunogen design based on the ebolavirus glycoprotein HR2-MPER region
Source: PLoS Pathog. 2022 May 18;18(5):e1010518. doi: 10.1371/journal.ppat.1010518 (PMC9170092; doi:10.1371/journal.ppat.1010518)
Supplement: S2 Table — (DOCX) [file ppat.1010518.s002.docx]

**S2 Table.** Sequences of immunogens carrying BDBV-MPER, BDBV-MPER-KPL, EBOV-MPER, SUDV-MPER, BDBV-MPER-I631V, BDBV-MPER-D624N and BDBV-MPER-KPL-D624N.

| >BDBV-MPER  FSAVVSVGDWLQAIKMDRYKDNFTAAGYTTLEAVRNMTLDDLARIGITAITHQNKIQDSIDQIIHDFIDMHG |
| --- |
| >BDBV-MPER-KPL  FSAVVSVGDWLQAIKMDRYKDNFTAAGYTTLEAVRNMTLDDLARIGITAITHQNKIQDSIDQIIHDFIDKPL |
| >EBOV-MPER  FSAVVSVGDWLQAIKMDRYKDNFTAAGYTTLEAVRNMTLDDLARIGITAITHQNKIQDSIDQIIHDFVDKTL |
| >SUDV-MPER  FSAVVSVGDWLQAIKMDRYKDNFTAAGYTTLEAVRNMTLDDLARIGITAITHQNKIQDSINQIIHDFINKPL |
| >BDBV-MPER-I631V  FSAVVSVGDWLQAIKMDRYKDNFTAAGYTTLEAVRNMTLDDLARIGITAITHQNKIQDSIDQIIHDFVDMHG |
| >BDBV-MPER-D624N  FSAVVSVGDWLQAIKMDRYKDNFTAAGYTTLEAVRNMTLDDLARIGITAITHQNKIQDSINQIIHDFIDMHG |
| >BDBV-MPER-KPL-D624N  FSAVVSVGDWLQAIKMDRYKDNFTAAGYTTLEAVRNMTLDDLARIGITAITHQNKIQDSINQIIHDFIDKPL |
