# Supplementary figures and images for: Epitope-focused immunogen design based on the ebolavirus glycoprotein HR2-MPER region
Source: PLoS Pathog. 2022 May 18;18(5):e1010518. doi: 10.1371/journal.ppat.1010518 (PMC9170092; doi:10.1371/journal.ppat.1010518)

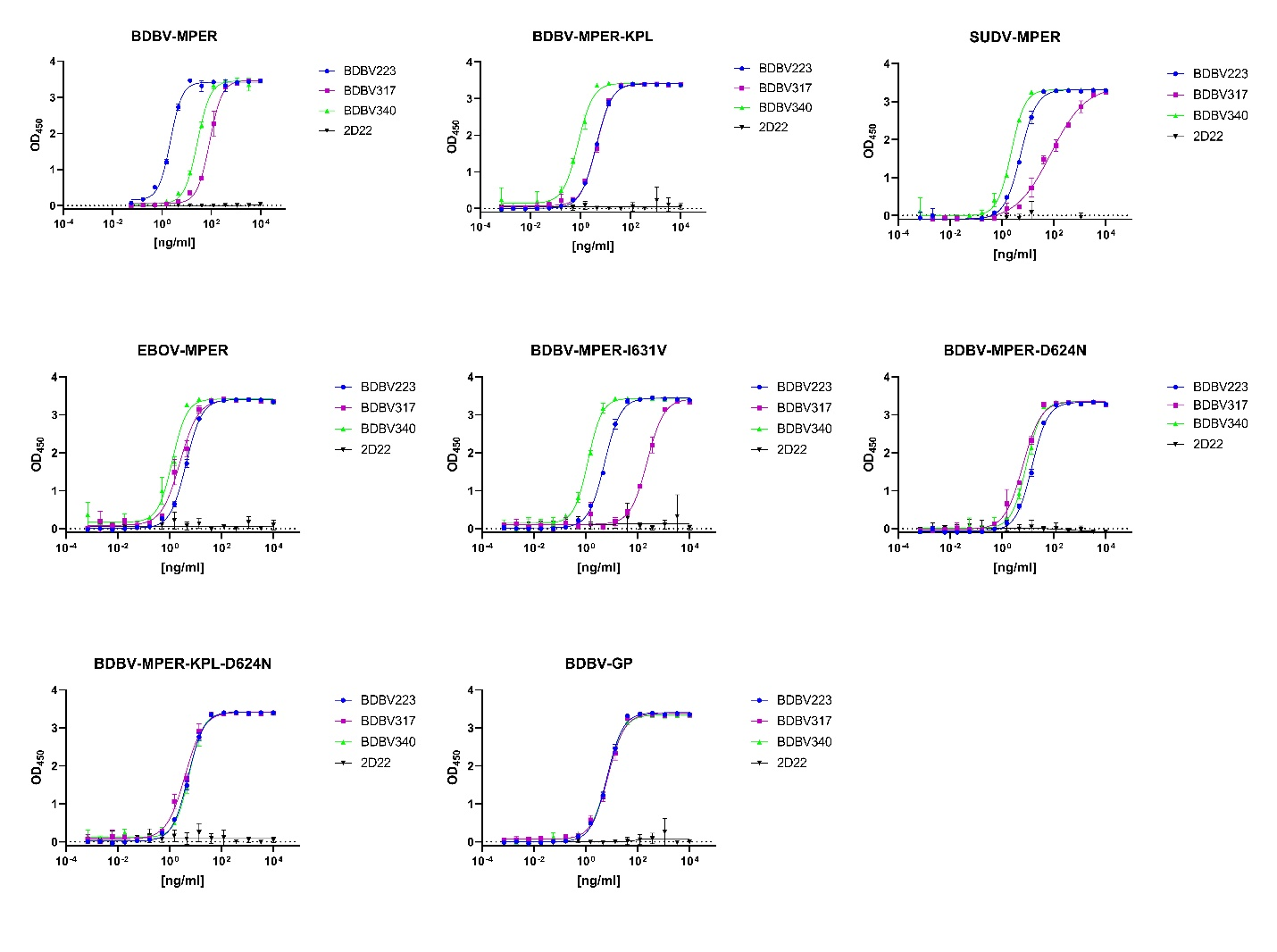

Supplement: S1 Fig — (TIF) [file ppat.1010518.s003.tif]

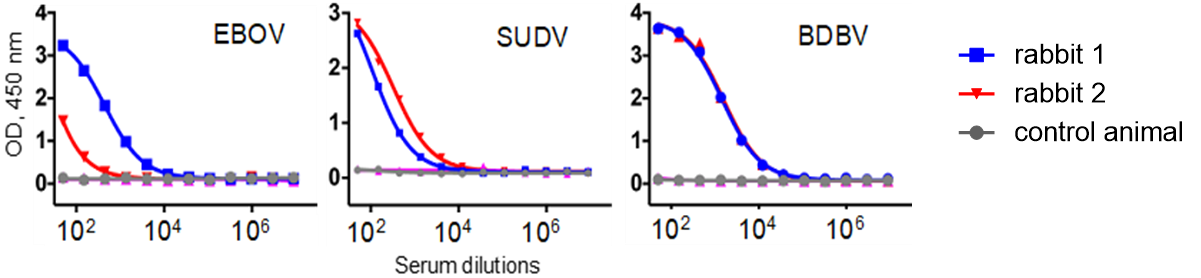

Supplement: S2 Fig — (TIF) [file ppat.1010518.s004.tif]

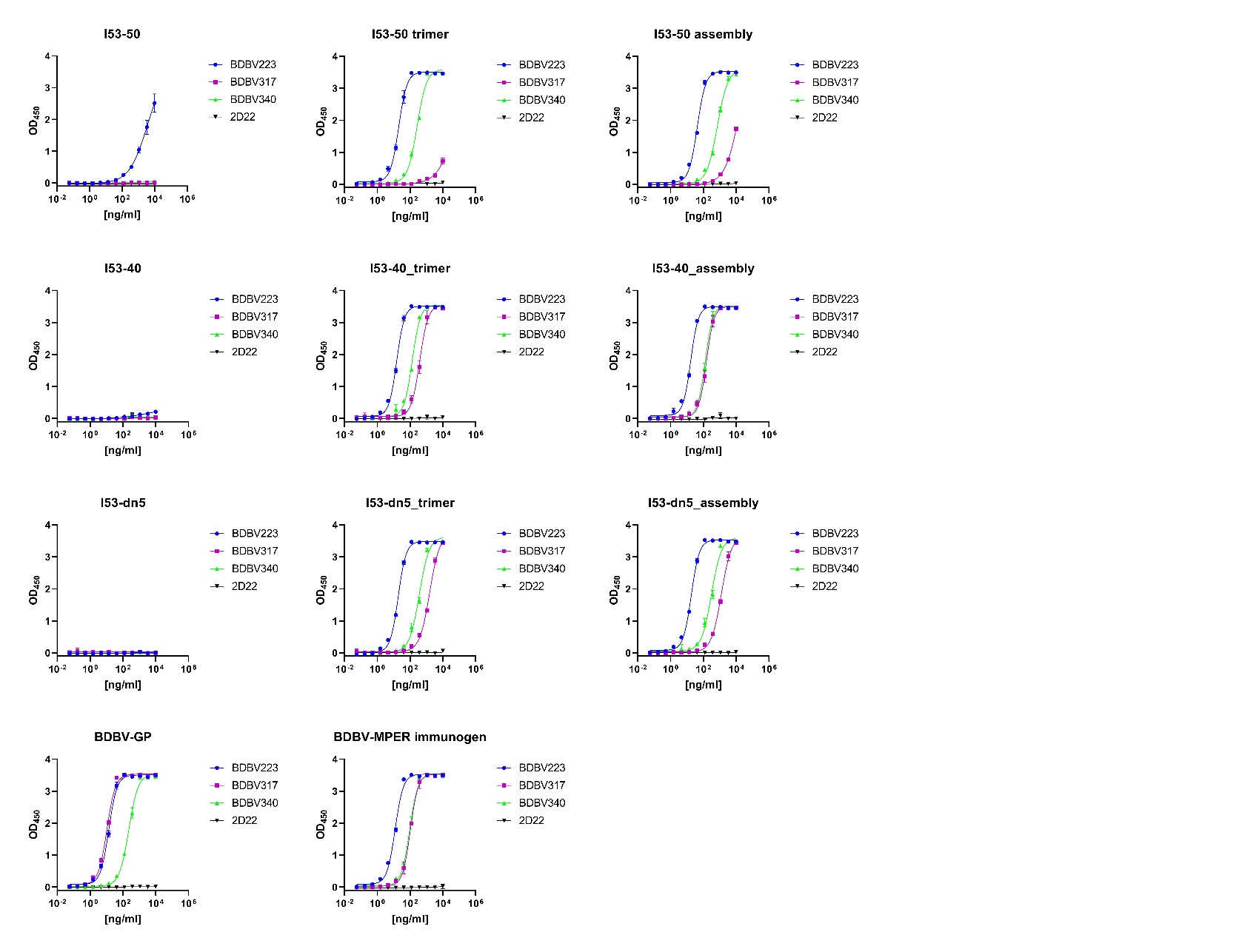

Supplement: S3 Fig — Nanoparticles displayed 60 copies of the BDBV-MPER immunogen on its surface. (TIF) [file ppat.1010518.s005.tif]

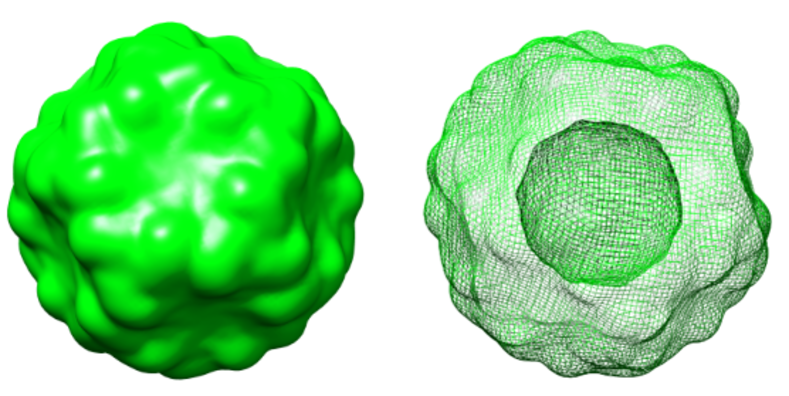

Supplement: S4 Fig — Notably, the immunogen cannot be resolved. (TIF) [file ppat.1010518.s006.tif]

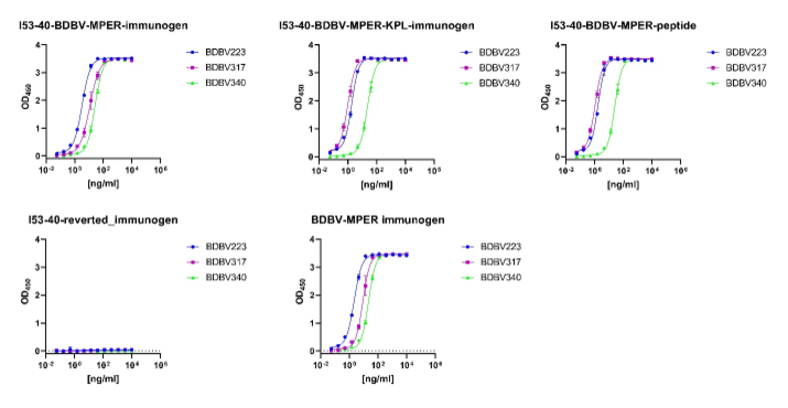

Supplement: S5 Fig — (TIF) [file ppat.1010518.s007.tif]

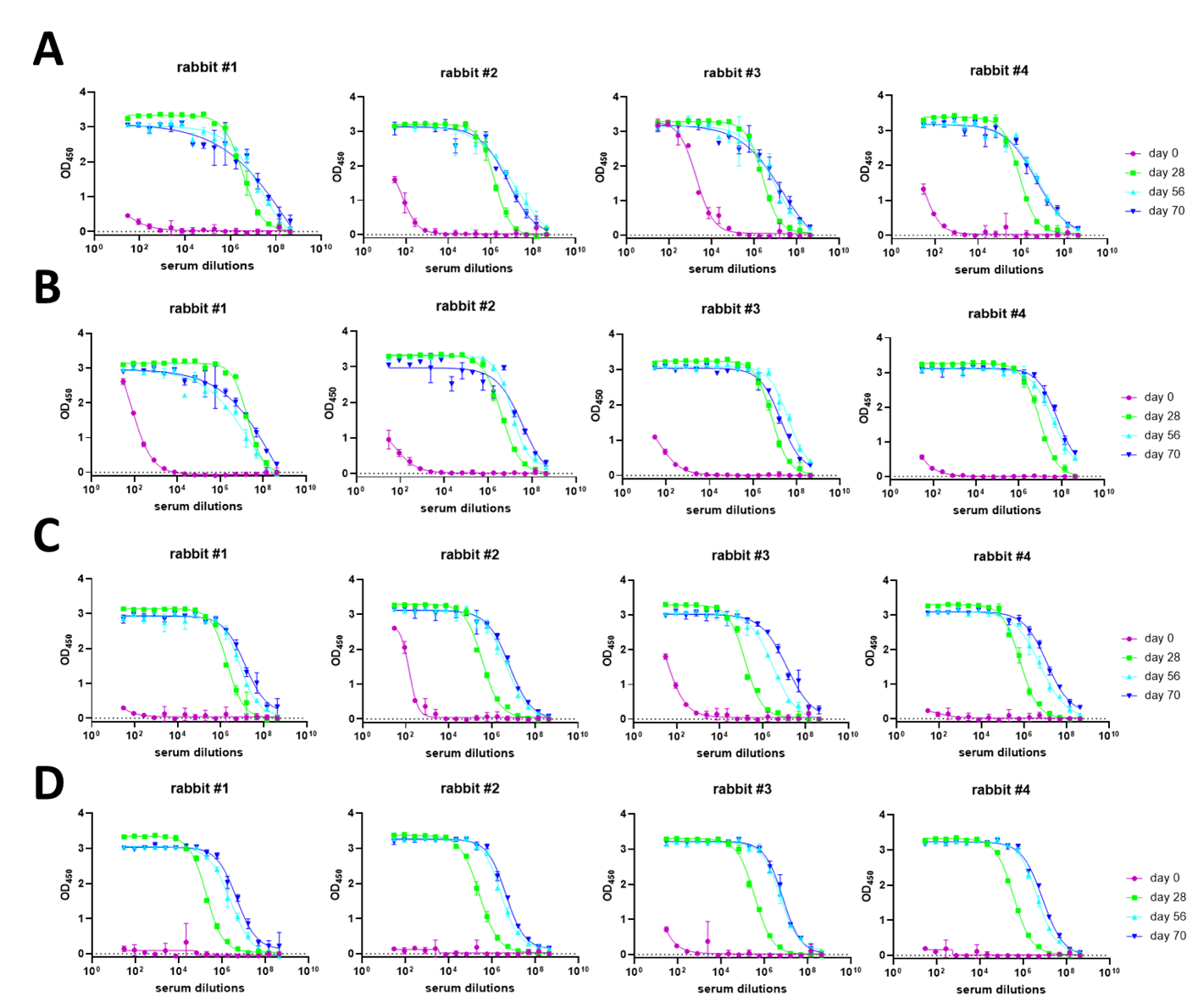

Supplement: S6 Fig — A. Serum binding of rabbits immunized with nanoparticle displayed BDBV-MPER immunogen to BDBV-MPER immunogen on nanoparticles. B. Reverted immunogen. C. BDBV-MPER-KPL immunogen. D. BDBV-MPER peptide. (TIF) [file ppat.1010518.s008.tif]

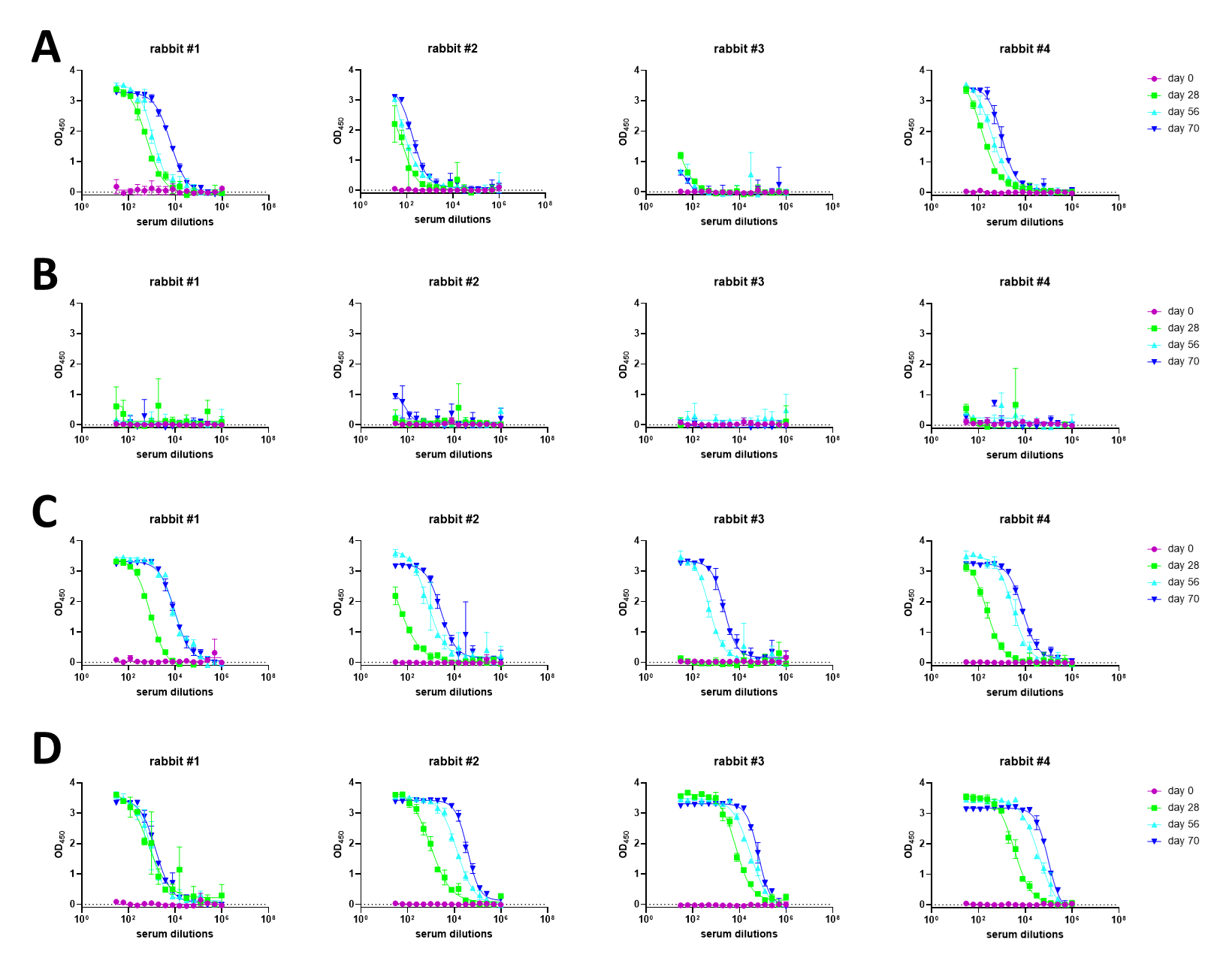

Supplement: S7 Fig — A. Serum binding of rabbits immunized with nanoparticle displayed BDBV-MPER immunogen to BDBV-MPER immunogen on nanoparticles. B. Reverted immunogen. C. BDBV-MPER-KPL immunogen. D. BDBV-MPER peptide. (TIF) [file ppat.1010518.s009.tif]

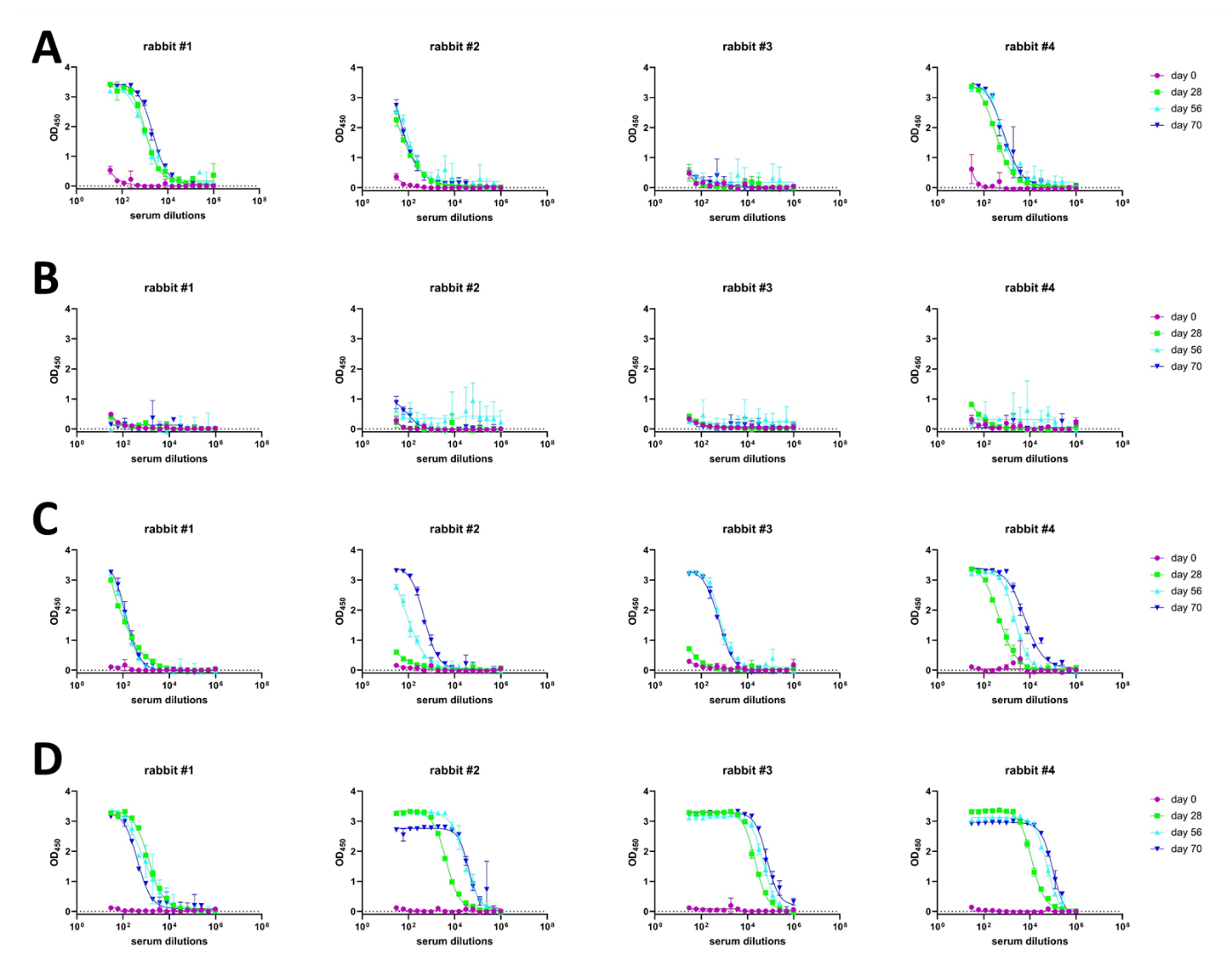

Supplement: S8 Fig — A. Serum binding of rabbits immunized with nanoparticle displayed BDBV-MPER immunogen to BDBV-MPER immunogen on nanoparticles. B. Reverted immunogen. C. BDBV-MPER-KPL immunogen. D. BDBV-MPER peptide. (TIF) [file ppat.1010518.s010.tif]

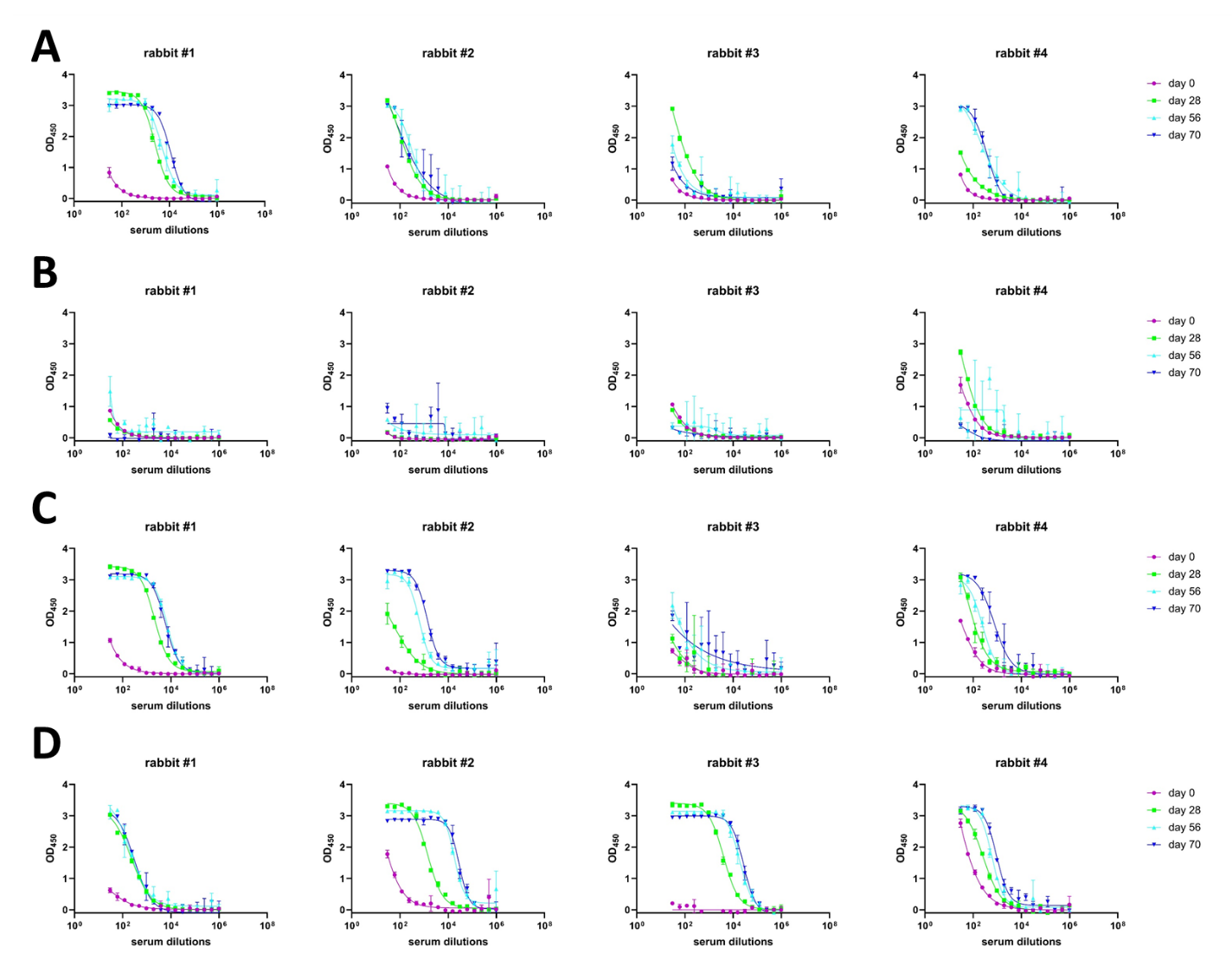

Supplement: S9 Fig — A. Serum binding of rabbits immunized with nanoparticle displayed BDBV-MPER immunogen to BDBV-MPER immunogen on nanoparticles. B. Reverted immunogen. C. BDBV-MPER-KPL immunogen. D. BDBV-MPER peptide. (TIF) [file ppat.1010518.s011.tif]

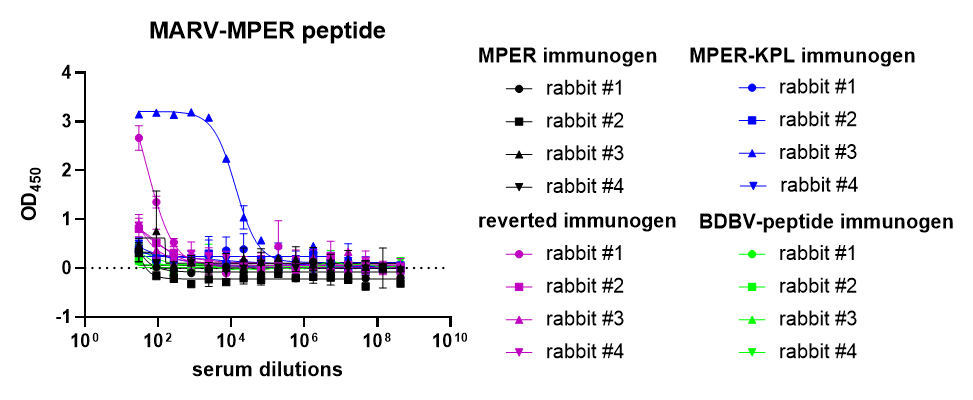

Supplement: S10 Fig — As MARV-MPER peptide, a peptide with the following sequence was used: GIEDLSRNISEQIDQIKKDEQKEG. (TIF) [file ppat.1010518.s012.tif]

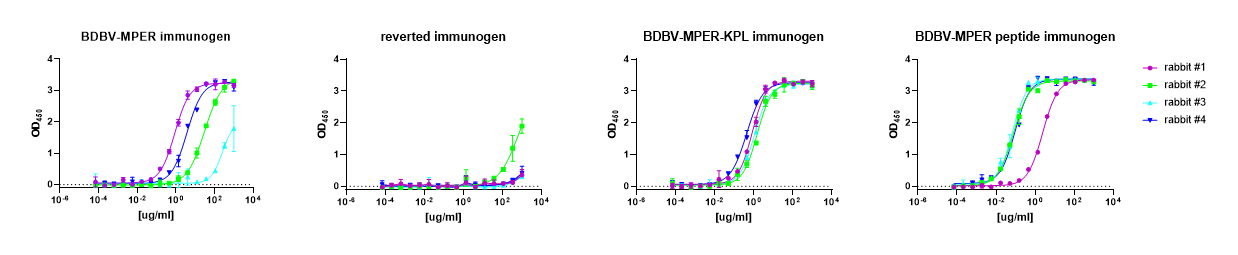

Supplement: S11 Fig — (TIF) [file ppat.1010518.s013.tif]

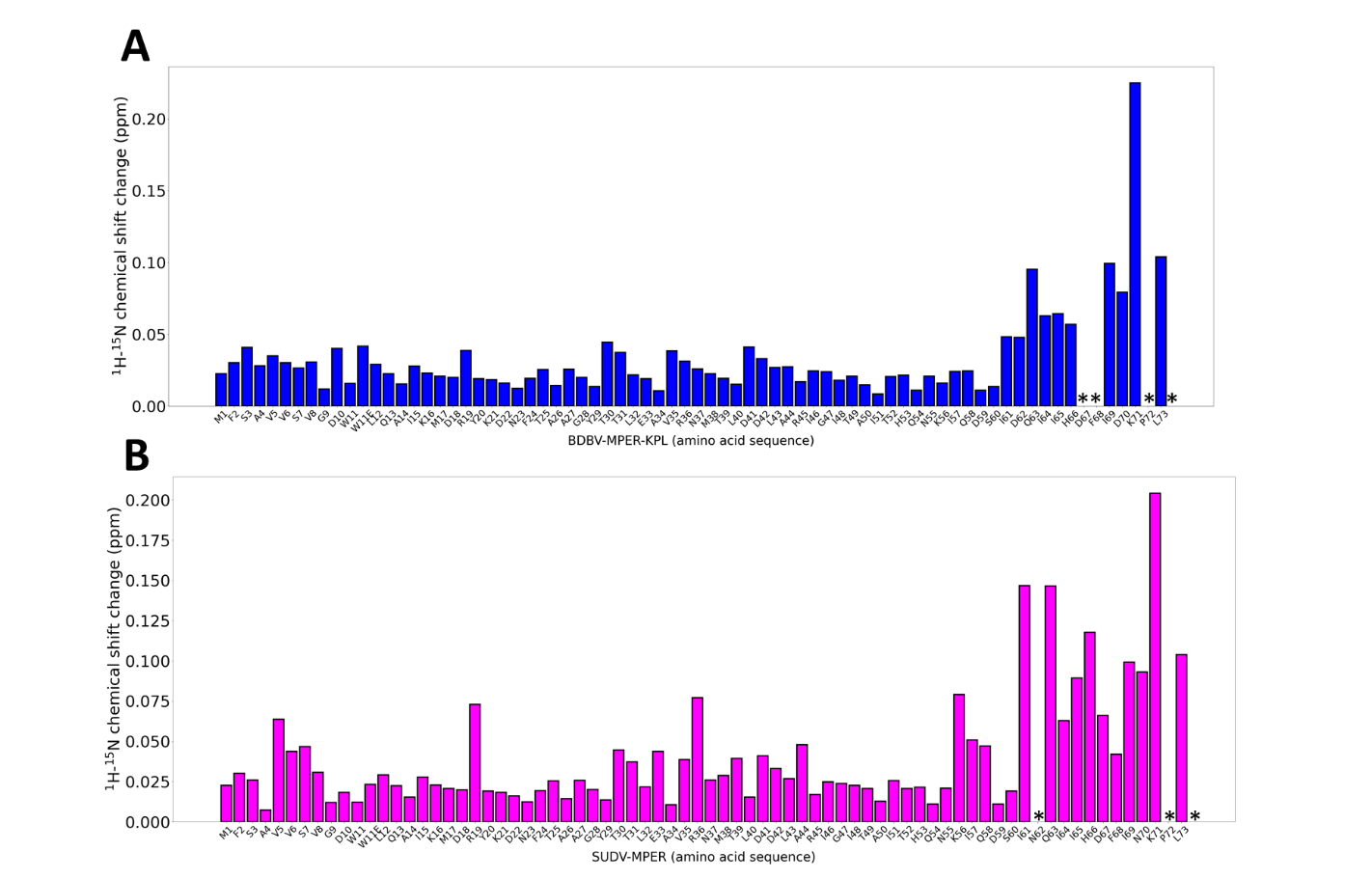

Supplement: S12 Fig — As a reference the BDBV-MPER immunogen was used. * indicates positions where a peak was not matched. A. Change in chemical shift plotted over the sequence of BDBV-MPER-KPL in comparison to the BDBV-MPER immunogen. B. Change in chemical shift plotted over the sequence of SUDV-MPER in comparison to the BDBV-MPER immunogen. (TIF) [file ppat.1010518.s014.tif]

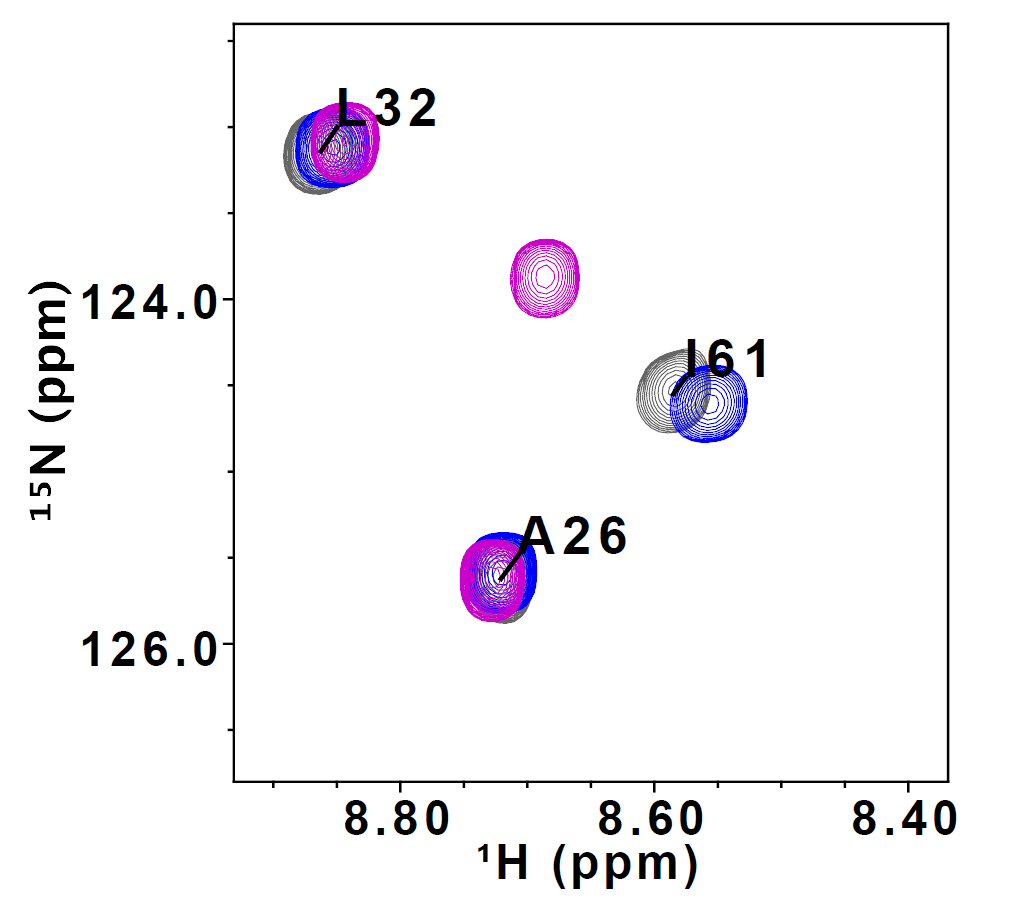

Supplement: S13 Fig — (TIF) [file ppat.1010518.s015.tif]

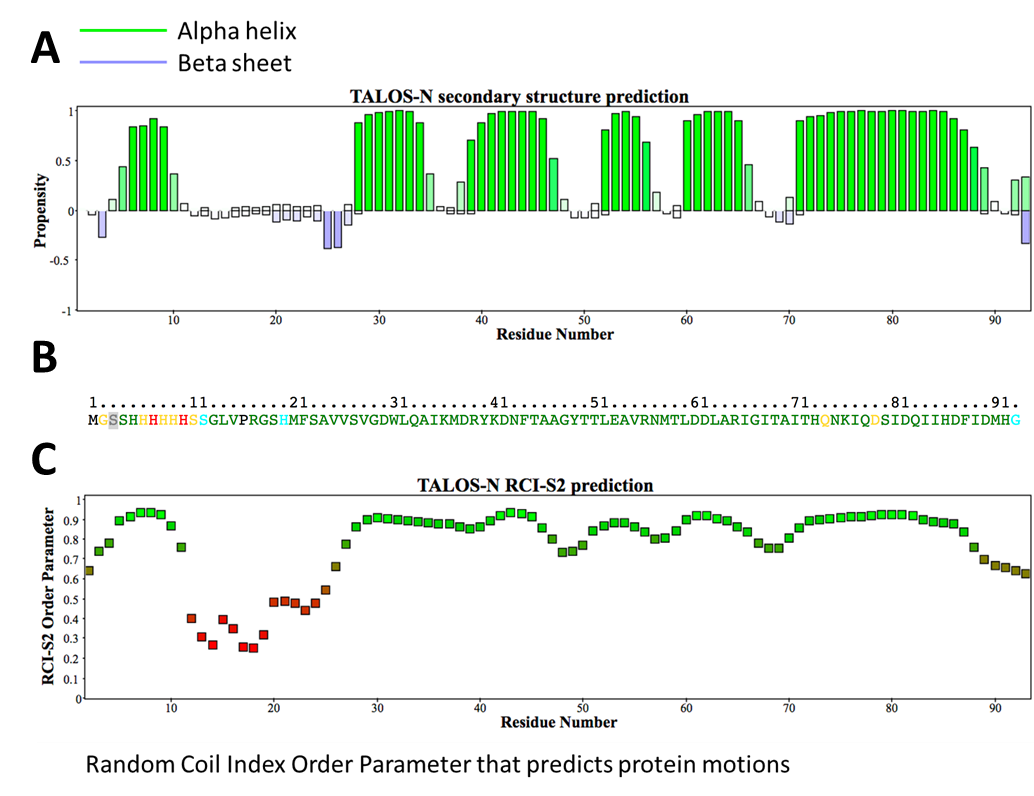

Supplement: S14 Fig — A. Prediction of secondary structure elements. Green indicates helical parts, light blue indicates β-sheets. B. Sequence of construct used for solution NMR experiments. C. Predicted unstructured areas have a low Random Coil Index Order Parameter. (TIF) [file ppat.1010518.s016.tif]

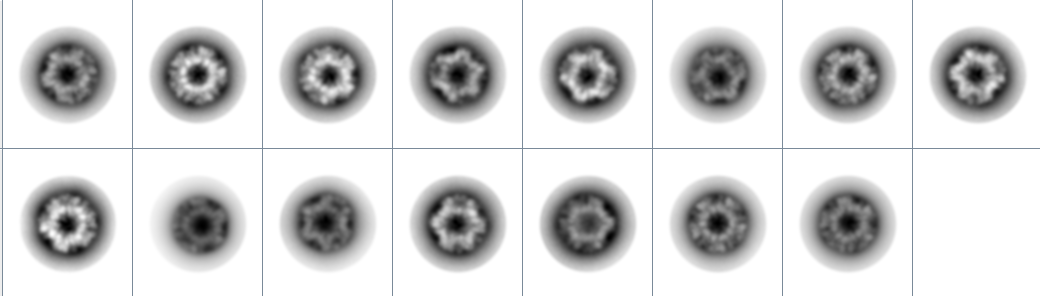

Supplement: S15 Fig — (TIF) [file ppat.1010518.s017.tif]

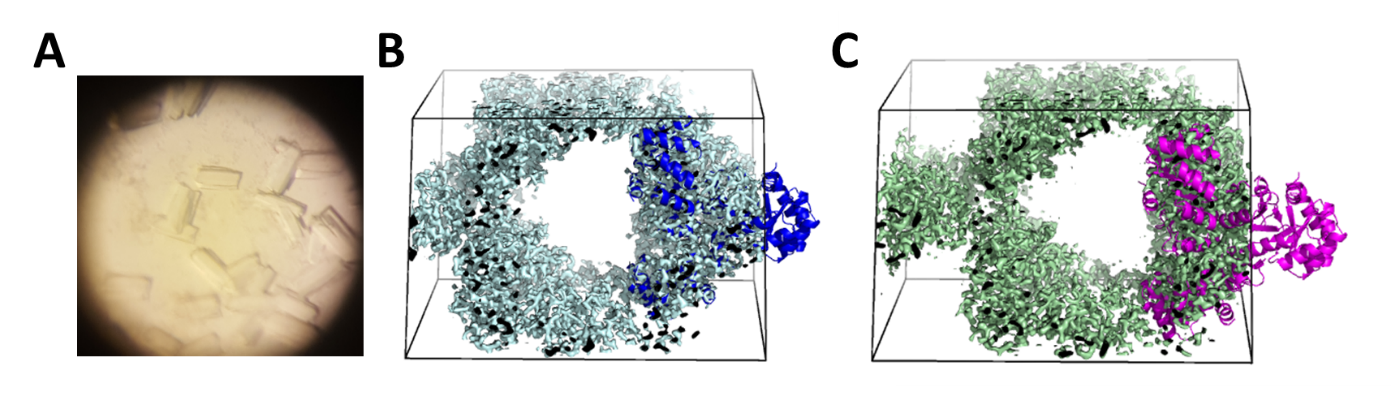

Supplement: S16 Fig — A. Crystals of I53-50 trimeric component with BDBV-MPER immunogen. Crystals are not formed homogenous. B. Unit cell of the density from I53-50 BDBV-MPER trimeric construct at a resolution of 2.20 Å. As search model the trimeric component as reported in PDB: 5IM5 was used. C. Unit cell of the density from I53-50 SUDV-MPER trimeric construct at a resolution of 2.48 Å. (TIF) [file ppat.1010518.s018.tif]
