# Supplementary material for: Epitope-focused immunogen design based on the ebolavirus glycoprotein HR2-MPER region
Source: PLoS Pathog. 2022 May 18;18(5):e1010518. doi: 10.1371/journal.ppat.1010518 (PMC9170092; doi:10.1371/journal.ppat.1010518)
Supplement: S1 Text — (DOCX) [file ppat.1010518.s019.docx]

**Computational protocol**

Side chain grafting and backbone grafting were executed as described in Silva *et al*. [1] Detailed protocols are available as part of the work from Silva *et al*. or a newer version is in included in the Rosetta workshop material from April 2019 [2]. We recommend using the most recent version of the reported protocols.

Input required for scaffold design:

1. Rosetta Software: Rosetta is a suite of programs for biomolecular modeling and design that can be downloaded from rosettacommons.org and is free for academic use [3, 4]. The Rosetta version used for this study was f66c697544177207b79cb60de513232df7a3f62c.

2. Starting structure with the epitope: Starting structure for the BDBV-MPER epitope was obtained from a structure of a BDBV-MPER peptide in complex with neutralizing antibody BDBV223 resolved at 3.7 Å (PDB-Id: 6n7j [5]). The motif TDKIDQIIHDFID used from BDBV-MPER contains residues 620-632 of the BDBV-GP sequence.

3. A scaffold library of 2,460 structures from the PDB (as of September 2016) was prepared based on the following criteria: (i) crystal structures with high resolution (< 2.5 Å), (ii) protein was reported to be expressed in *E. coli*, (iii) has a single protein chain in its asymmetric unit, (iv) no bound ligands or modified residues. All 2,460 structures were relaxed with Rosetta by constraining the protein backbone to its start coordinates [6].

Three protocols were subsequently applied to the input:

*Side Chain Grafting*. The scaffold library was computationally scanned for possible graft sites. This approach leads to minimal changes in the scaffold sequence leading to higher probability of correctly folded designs. However, surrounding residues on the scaffold that interacts with the context are designed for better interactions. The BDBV223 structure was used as the context and as motif was the 13 residue long alpha-helical structure of the BDBV-MPER peptide in the co-crystal structure defined (residue number 620-632) that are in direct contact with the binding antibody. Side chain grafting was performed using a Rosetta XML script as described in Silva *et al*. [1] The algorithm was allowed to shorten the motif by one residue on the N-terminal side during the alignment process along with full-length motif alignment onto the scaffold library backbones. After side chain grafting, scaffold residues within 8 Å of the context residues are allowed to be designed while the hotspot residues and context residues can only change rotamer conformations. One more round of design was performed after rigid body minimization of the complex. A set of 258 protein scaffolds qualified for transplanting the motif onto them. After analyzing the shape complementarity, ddG, graft_RMSD, buried unsatisfied hydrogen-bonding atoms in the interface and manual inspection of the resulting 258 grafts, five scaffolds (PDB-IDs: 1b0x [7], 3o3k [8], 4g3o [9], 4o7q [10] and 4o9f [11]) were selected with protein sequences shorter than 100 residues. Further rounds of design after manual inspection resulted in the redesign of two methionine residues in 1B0X to abolish 1B0X dimerization, as observed in the 1b0x crystal structure. Short-listed designs were checked for their foldability using *de novo* folding of the designed sequences. Seven designs, 1b0x_0001_0001, 1b0x_0001_0002, 3o3k_0001_0001, 4g3o_0001_0001, 4g3o_0001_0001, 407q_0001_0001 and 4o9f_0001_0001, from side chain grafting based on five different scaffolds were experimentally tested for their in vitro expression from *E. coli*.

*Backbone Grafting*. The scaffold search is rather different for backbone grafting as any secondary structure even with different length of amino acids can replace the scaffold backbone based on the N- and C-terminus alignment. Three designs steps involved in backbone grafting cover the design of scaffold residues within a hydrophobic core of up to 15 Å away from the interface, design of core and boundary residues that are up to 12 Å from the interface and optimization of all the scaffold residues that are 8 Å from the context surface. Initial screening of 2,459 scaffolds with MotifGraft_bb.xml (as explained in protocol capture) resulted in a grafting the 13 residues long alpha-helix motif onto 997 scaffolds. In many designs, helical segments of the scaffold were replaced by helical motif but in other scaffolds a non-helical part of the structure was replaced by the motif. Designs were filtered based on binding energy, high shape complementarity and low number of buried unsatisfied hydrogen bond atoms and manual inspection. MultiplePoseMover was used for backbone grafting of the helical motif onto scaffolds: 1b0x [7], 2qvo [12], 3o3k [8] and 4g3o [9] to generate more designs. Two designs from 1b0x scaffold were tested for potential folding ability using *de novo* folding (1b0x_0001_0003.pdb and 1b0x_0001_0005.pdb). Three designs were selected from the backbone grafting approach for *in vitro* protein expression: 1b0x_0001_0003, 3o3k_0001_0002 and 4g3o_0001_0003.

*FoldFromLoops*. (Note that FoldFromLoops has been replaced by FunFolDes [13], a newer XML-executable version of the protocol, that now should be used instead of FoldFromLoops). In this method, the user defines the segment in the template protein, which will be replaced by the functional motif, which in our case is the alpha-helix of the BDBV-MPER peptide The final scaffold design is restrained to the same three–dimensional structure as template protein even though the sequence can be totally redesigned [14]. Only one design was selected from this method, using 1b0x as scaffold. All resulting designs were selected based on Rosetta total score, RMSD to the template protein structure, binding affinity to BDBV223, shape complementarity, number of unsaturated H-bonds and manual inspection in PyMOL.

In total, eleven designs based on five different starting scaffolds with amino acid lengths < 100 were tested for expression and binding affinity to BDBV223. Seven among them were from side chain grafting, three from backbone grafting and one from FoldFromLoops. 4g3o_0001_0001, 3o3k_0001_0002 and 3o3k_0001_0001 did not express. 4g3o_0001_0002 and 1b0x_0001_0001 expressed but poorly. ffl-1b0x_0165, 4o9f_0001_0001, 4o7q_0001_0001 expressed. 1b0x_0001_0002 expressed very well. 4g3o_0001_0003, 1b0x_0001_0003, ffl-1b0x_0165, 1b0x_0001_0002, 4o9f_0001_0001 and 4o7q_0001_0001 designs were tested for binding to BDBV223. 1b0x_0001_0002 bound to BDBV223, BDBV340 and BDBV317 as well. 1b0x_0001_0001 did not show any binding, with 1b0x_0001_0002 being the optimized version of 1b0x_0001_0001 where two methionine residues were mutated to other hydrophobic residues because they are known to cause dimerization of the Eph SAM domain.

**References**

1. Silva DA, Correia BE, Procko E. Motif-Driven Design of Protein-Protein Interfaces. Methods Mol Biol. 2016; 1414:285-304.

2. Schoeder CT, Schmitz S, Adolf-Bryfogle J, Sevy AM, Finn JA, Sauer MF, et al. Modeling Immunity with Rosetta: Methods for Antibody and Antigen Design. Biochemistry. 2021; 60(11):825-46.

3. Leman JK, Weitzner BD, Lewis SM, Adolf-Bryfogle J, Alam N, Alford RF, et al. Macromolecular modeling and design in Rosetta: recent methods and frameworks. Nat Methods. 2020; 17(7):665-80.

4. Leaver-Fay A, Tyka M, Lewis SM, Lange OF, Thompson J, Jacak R, et al. ROSETTA3: an object-oriented software suite for the simulation and design of macromolecules. Methods in enzymology. 2011; 487:545-74.

5. King LB, West BR, Moyer CL, Gilchuk P, Flyak A, Ilinykh PA, et al. Cross-reactive neutralizing human survivor monoclonal antibody BDBV223 targets the ebolavirus stalk. Nat Commun. 2019; 10(1):1788.

6. Nivon LG, Moretti R, Baker D. A Pareto-optimal refinement method for protein design scaffolds. PLoS One. 2013; 8(4):e59004.

7. Stapleton D, Balan I, Pawson T, Sicheri F. The crystal structure of an Eph receptor SAM domain reveals a mechanism for modular dimerization. Nat Struct Biol. 1999; 6(1):44-9.

8. Zhu JY, Fu ZQ, Chen L, Xu H, Chrzas J, Rose J, et al. Structure of the Archaeoglobus fulgidus orphan ORF AF1382 determined by sulfur SAD from a moderately diffracting crystal. Acta Crystallogr D Biol Crystallogr. 2012; 68(Pt 9):1242-52.

9. Crystal structure of the CUE domain of the E3 ubiquitin ligase AMFR (gp78). 2012.

10. Lu A, Kabaleeswaran V, Fu T, Magupalli VG, Wu H. Crystal structure of the F27G AIM2 PYD mutant and similarities of its self-association to DED/DED interactions. J Mol Biol. 2014; 426(7):1420-7.

11. Xu H, He X, Zheng H, Huang LJ, Hou F, Yu Z, et al. Structural basis for the prion-like MAVS filaments in antiviral innate immunity. Elife. 2014; 3:e01489.

12. Crystal structure of AF1382 from Archaeoglobus fulgidus. 2007.

13. Bonet J, Wehrle S, Schriever K, Yang C, Billet A, Sesterhenn F, et al. Rosetta FunFolDes - A general framework for the computational design of functional proteins. PLoS Comput Biol. 2018; 14(11):e1006623.

14. Correia BE, Bates JT, Loomis RJ, Baneyx G, Carrico C, Jardine JG, et al. Proof of principle for epitope-focused vaccine design. Nature. 2014; 507(7491):201-6.
