# Supplementary material for: Epitope-focused immunogen design based on the ebolavirus glycoprotein HR2-MPER region
Source: PLoS Pathog. 2022 May 18;18(5):e1010518. doi: 10.1371/journal.ppat.1010518 (PMC9170092; doi:10.1371/journal.ppat.1010518)
Supplement: S2 Text — (DOCX) [file ppat.1010518.s020.docx]

**Supplemental Methods**

**Crystallography**

Protein solution buffer was changed for a buffer containing 10 mM Tris, 2 mM TPEC, pH 8.0 and concentrated to 10 mg/ml. Automated crystallization trials were set-up with a Mosquito® crystal pipetting robot (SPT Labtech, Melbourn, UK) in a 1:1 dilution with the protein sample and stored at room temperature in a RockImager® (Formulatrix, Bedford, MA, USA). Crystals formed after 10 days in the following buffer conditions: 25 mM Tris, 30% w/v PEG400 and 0.2 M MgCl_2_. Follow-up studies were performed in 24-well plate hanging drop or sitting drop crystallization plates (Hampton) titrating the conditions through 2% increment adjustments to the PEG400 concentration. Crystals formed in 26%, 28%, 30% (w/v) PEG400. As cryoprotection 20% glycerol was added to the initial buffer conditions and crystals transferred into liquid nitrogen. X-ray diffraction data were collected at Advanced Photon Source LS-CAT beamline 21-ID-G or -F.
